# Quantifying MR head motion in the Rhineland Study – A robust method for population cohorts

**DOI:** 10.1016/j.neuroimage.2023.120176

**Published:** 2023-05-18

**Authors:** Clemens Pollak, David Kügler, Monique M.B. Breteler, Martin Reuter

**Affiliations:** a AI in Medical Imaging, German Center for Neurodegenerative Diseases (DZNE), Bonn, Germany; b Population Health Sciences, German Center for Neurodegenerative Diseases (DZNE), Bonn, Germany; c Institute for Medical Biometry, Informatics and Epidemiology (IMBIE), Faculty of Medicine, University of Bonn, Bonn, Germany; d A.A. Martinos Center for Biomedical Imaging, Massachusetts General Hospital, Boston, MA, USA; e Department of Radiology, Harvard Medical School, Boston, MA, USA

**Keywords:** 0000, 1111, MRI Head tracking, Population study, Depth camera, 0000, 1111

## Abstract

Head motion during MR acquisition reduces image quality and has been shown to bias neuromorphometric analysis. The quantification of head motion, therefore, has both neuroscientific as well as clinical applications, for example, to control for motion in statistical analyses of brain morphology, or as a variable of interest in neurological studies. The accuracy of markerless optical head tracking, however, is largely unexplored. Furthermore, no quantitative analysis of head motion in a general, mostly healthy population cohort exists thus far. In this work, we present a robust registration method for the alignment of depth camera data that sensitively estimates even small head movements of compliant participants. Our method outperforms the vendor-supplied method in three validation experiments: 1. similarity to fMRI motion traces as a low-frequency reference, 2. recovery of the independently acquired breathing signal as a high-frequency reference, and 3. correlation with image-based quality metrics in structural T1-weighted MRI. In addition to the core algorithm, we establish an analysis pipeline that computes average motion scores per time interval or per sequence for inclusion in downstream analyses. We apply the pipeline in the Rhineland Study, a large population cohort study, where we replicate age and body mass index (BMI) as motion correlates and show that head motion significantly increases over the duration of the scan session. We observe weak, yet significant interactions between this within-session increase and age, BMI, and sex. High correlations between fMRI and camera-based motion scores of proceeding sequences further suggest that fMRI motion estimates can be used as a surrogate score in the absence of better measures to control for motion in statistical analyses.

## 1. Introduction

Magnetic resonance imaging (MRI) of the brain is recognized as the gold-standard for in-vivo analysis of the central nervous system, its organisation, function, and diagnosis. The advent of high-quality structural 3D MR acquisition and sensitive software tools for its morphometric analysis have enabled accurate quantification of subtle neuroanatomical changes (Fischl, 2012; Henschel et al., 2022; Jenkinson et al., 2012; Reuter et al., 2012), e.g. in large cohort studies and clinical trials. While these neuromorphometric association studies commonly control for confounding variables and demographics, they frequently omit potential confounders of the MR acquisition (such as sequence, hardware, or firmware differences). This is especially problematic if confounding variables correlate with variables of interest and may bias statistical analysis. One such confounder is head motion. Head motion has been shown to systematically bias MRI-based neuromorphometric analysis (Alexander-Bloch et al., 2016; Biller et al., 2015; Gilmore et al., 2021; Nakamura et al., 2014; Pardoe et al., 2016; Reuter et al., 2015; Rosen et al., 2018; Savalia et al., 2017; Streitbürger et al., 2012). Specifically, motion-induced imaging artefacts can result in spurious findings of reduced gray matter volume estimates in T1-weighted MRI analysis (Alexander-Bloch et al., 2016; Gilmore et al., 2021; Pardoe et al., 2016; Reuter et al., 2015; Rosen et al., 2018; Savalia et al., 2017), even when they are barely visually detectable. Such motion induced biases are expected to increase with the introduction of higher resolution imaging sequences (Stucht et al., 2015) and extended scan times per sequence. Moreover, increased head motion is often correlated with variables of interest such as age (Madan, 2018; Savalia et al., 2017), body mass index (BMI) (Beyer et al. (2017, 2020), and various neurological disorders including autism (Cox et al., 2017; Dosenbach et al., 2017; Torres and Denisova, 2016), Parkinson’s (Thenganatt and Jankovic, 2016), Alzheimer’s (Versluis et al., 2010), and Huntington’s disease (Rizk-Jackson et al., 2011), potentially resulting in an overestimation of these effects.

Common practice, such as the attempts to reduce motion by verbal instructions and cushioning of participants during acquisition, are highly recommended, yet, cannot completely control head motion. Retrospective expert grading of motion, based on imaging artifacts, can help identify severe motion cases, leading to their exclusion, but will not address consistent biases induced by small head motion. In contrast, the explicit inclusion of motion measurements as a control variable in statistical models offers a way to control even small motion biases and can help to disentangle motion effects from real anatomical changes. This, however, requires an accurate measurement of motion during MR acquisition in the first place.

In order to address this demand, we propose a *robust registration method* and an aggregated *motion score* for highly accurate head tracking and motion quantification during MR acquisition via a commercially available depth camera. For this, our registration yields a time series of head poses (*motion trace*) by robustly registering individual point cloud data derived from depth video frames to a reference frame. The resulting motion trace is highly accurate and able to recover high-frequency details. Our method significantly outperforms the vendor-supplied head tracking method (Slipsager et al. 2019, 2022) on three novel evaluations: 1. increased similarity to functional MRI motion estimates, 2. larger mutual information with a high frequency respiratory measurements, and 3. improved correlation of our motion score with various image quality metrics.

Finally, we apply our method in the Rhineland Study (Breteler et al., 2014; Stöcker, 2016), which is the first time optical tracking is used in a large population cohort study. While other work focuses on datasets with high motion levels, our method tackles the head tracking task in a cohort from the general population, where motion levels are low (requiring high sensitivity) and tracking needs to work on hundreds of participants with minimal intervention. We use our method to confirm previously known correlations of BMI and age with motion in the Rhineland Study. Additionally, we establish, that head motion increases over the course of the scan session, and that motion measured from functional MRI (fMRI) acquisitions can serve as a reasonable proxy of motion in adjacent scans.

### 1.1. Head motion estimation

As previously motivated, neuroimaging studies require quantitative head motion estimates to statistically model motion-induced biases and correct for correlates of increased head motion. These motion estimates can either be obtained during acquisition or retrospectively estimated from acquired images (Pollak et al., 2023). In practice, head motion is a composite of different motion patterns ranging from abrupt, large movements to slow drift and periodical patterns such as breathing (Zaitsev et al., 2015). To capture all types of motion (including small and rapid motion), a head tracking with high accuracy and high frequency is required. Coughing and diseases related burst movements can occur within a fraction of a second (Dbouk and Drikakis, 2020) or milliseconds (Eberhardt and Topka, 2017) and might not be detectable in motion tracking with sampling rates at 2 Hz or below.

Multiple recording and processing paradigms have been established for head tracking. They either directly utilize the MRI scanner or introduce external devices and are subject to individual trade-offs with respect to ease of use, accuracy, and sampling frequency. The most widespread approach is to register and align subsequent images (frames) of fMRI sequences. While its primary purpose is to correct inter-frame motion to establish frame-to-frame correspondences (Goto et al., 2015; Jenkinson et al., 2002; Maknojia et al., 2019), individual frame-to-frame alignments yield low-frequency motion estimates for the time frame of the fMRI sequence. Depending on its specific acquisition parameters, individual frames are acquired over 0.5 to several seconds aggregating the head motion across the frame and limiting the sampling rate and ability to detect short bursts of motion. Unfortunately, the acquisition and alignment of fMRI images itself is also affected by motion artifacts induced by intra-frame motion, resulting in unreliable estimates for rapid motion (Maknojia et al., 2019). Finally, the multi-frame nature of the approach makes it inapplicable to singleframe MRI modalities, such as T1-weighted (T1w) structural scans and others. Based on the assumption of similar motion between functional scans and adjacent acquisitions, the fMRI-based motion estimate is sometimes used as a proxy for motion in adjacent structural scans (Pardoe et al., 2016; Savalia et al., 2017). While it has been shown, that ranking participants fMRI-based motion estimates is stable over multiple fMRI sequences in the same session, a direct comparison with motion during structural imaging sequences has not been performed in previous work.

Alternative MR scanner-based approaches rely on dedicated navigator sequences or signals that can be divided into three categories “FID”, “k-space”, and “image-based ” navigators (Andronesi et al., 2021). FID navigators (Kober et al., 2011) estimate motion from sensitivity profiles of multiple coils rapidly. However, the methods require calibration or modeling for quantitative tracking and have limited resolution in the acquired data (Andronesi et al., 2021). K-space navigators, like navigator echoes (Costa et al., 2005; Fu et al., 1995), are used to perform real-time and inter-scan motion correction (Andronesi et al., 2021), which resulted in multiple methods (Van der Kouwe et al., 2006; Welch et al., 2002) with varying trade-offs (Andronesi et al., 2021) between accuracy and acquisition time. Image-based navigators acquire small volume frames, such as echo planar sequences (EPI) (Alhamud et al., 2012; Hess et al., 2011; Tisdall et al., 2012) that are acquired interleaved with other modalities and can be used to estimate motion via image registration. This estimation method is limited by the low image resolution and frequency of frames, and can increase the overall scan time (Andronesi et al., 2021). None of the scanner-based methods are currently widely used and scanner manufacturers move toward supporting external optical tracking systems (Andronesi et al., 2021), due to their higher sampling rates and increased accuracy.

Approaches based on optical tracking commonly detect the motion of markers, which are either fixed to the skin (Forman et al., 2011; Maclaren et al., 2012) or integrated into a dental brace (Maclaren et al., 2012; Stucht et al., 2015; Todd et al., 2015). Since optical systems operate independently of the scanner, they can capture high-frequency estimates for all MRI modalities without requiring integration into the acquisition protocol – while providing higher frequency and lower latency estimates than navigators (Aksoy et al., 2017). However, marker attachment may result in increased motion (e.g. saliva build up due to dental brace (Maclaren et al., 2012)) and longer scan preparation, which limits their applicability in high-throughput settings (Herbst et al., 2013).

To mitigate this limitations, recent work introduced markerless tracking of portions of the face with external cameras (Kyme et al., 2020; Olesen et al., 2010; Pardoe et al., 2021; Slipsager et al., 2019). These methods use high quality cameras e.g. time of flight cameras that capture depth images, to infer the head pose. While markerless tracking represents an unobtrusive tracking paradigm, it is more susceptible to challenges posed by participant-specific features, e.g. facial hair, head size, proximity to camera, and eye or skin motion from facial expressions.

The only available software that enables motion tracking based on 3D face captures is the camera vendor software “TracSuite” (Bergström and Edlund, 2014). In addition to scanner-based and optical tracking methods, EEG (Laustsen et al., 2022) and sensing pads (Musa et al., 2022) have very recently been proposed for within-scanner high-frequency head motion tracking. These approaches are promising as they are not affected by facial expressions and do not require marker attachment. Their tracking accuracy, however, is not yet well established.

Generally, the lack of standardized evaluation metrics and experimental procedures limits the comparability of approaches across publications (Kyme et al., 2020; Laustsen et al., 2022; Maclaren et al., 2012; Musa et al., 2022; Pardoe et al., 2021; Slipsager et al., 2019; Todd et al., 2015). Especially prominent marker-based (Maclaren et al., 2012) and markerless (Slipsager et al., 2019) optical tracking methods are primarily validated with MRI motion correction capabilities, which makes comparison challenging. Besides different sequences and correction algorithms, the cohorts vary drastically from diseased pediatric patients (Slipsager et al., 2019) to healthy adults with extensive MRI experience (Maclaren et al., 2012).

On the other hand validations on phantoms with induced motion (Einspänner et al., 2022) are repeatable, and can be adjusted for varying motion sizes and patterns. These movable phantoms, however, are not widely available and currently unable to model all aspects relevant to motion tracking, like non-rigid facial expressions or the wide range of motion types (such as breathing, swallowing, coughing, drift). Human studies with induced/requested motion can be used to study strong head motion for method development. But these studies lack both repeatability and realistic motion levels – making across study comparisons extremely challenging. Unlike phantom studies, human studies can not be used for direct validation as the real motion (ground truth) is unknown. Due to these challenges, many head tracking approaches are not well validated, highlighting the need for multiple secondary metrics to test the validity of within-scanner head tracking approaches for human studies.

### 1.2. Head motion as a variable of interest

Beyond the confounding effect of motion on morphological analyses, head motion in the MRI scanner is subject to numerous research that aims to uncover the relationship between increased head movement and other biological markers. Motion has been shown to correlate with measures of impulsivity/hyperactivity, diabetes, hypertension, nicotine, alcohol use (Couvy-Duchesne et al., 2016; Ekhtiari et al., 2019; Hodgson et al., 2017; Kong et al., 2014; Wylie et al., 2014), and various neurological disorders (Cox et al., 2017; Rizk-Jackson et al., 2011; Thenganatt and Jankovic, 2016; Torres and Denisova, 2016). A prominent correlate of within-scan motion is age, where head motion is increased in younger pediatric cohorts (Afacan et al., 2016; Barkovich et al., 2019; Dosenbach et al., 2017; Janos et al., 2019; Oztek et al., 2020), but decreased in younger adult cohorts (Andrews Hanna et al., 2007; Chan et al., 2014; Madan, 2018; Pardoe et al., 2016; Savalia et al., 2017). Another strong correlate of within-scanner motion is the body mass index (BMI) (Beyer et al. (2017, 2020); Couvy-Duchesne et al., 2014; Ekhtiari et al., 2019; Hodgson et al., 2017; Kong et al., 2014) with a recent longitudinal study even showing the causality between weight loss and reduced within-scan motion in obese adults (Beyer et al., 2020).

On top of these correlates of in-scanner head motion, multiple independent studies (Couvy-Duchesne et al., 2014; Engelhardt et al., 2017; Hodgson et al., 2017) established in-scanner head motion as a heritable trait. Zeng et al. (Zeng et al., 2014) suggest that the association of reduced long-range connectivity and head motion is partially explained by individual variability in functional organization, in addition to bias due to motion artifacts, which outlines the previously discussed entanglement of cause and effect of motion in MRI acquisitions.

### 1.3. Scoring image quality and head motion

To correct for motion in statistical models and to model the relationship of motion and disease, we desire a scalar motion score during MRI acquisitions. Previously, manual motion scores where obtained by eyeballing image artifacts, such as ringing or blurring, that may be caused by excessive motion during the scan. However, such visual inspection cannot reliably identify all motion artifacts affecting downstream analyses as motion effects can still be detected in high quality images that pass visual quality checks (Pollak et al., 2023; Reuter et al., 2015). The Rhineland Study performs visual quality checks for all T1w images, with three possible ratings: (PASS, WARNING, FAIL). In the subset of images used in this work, none failed and only 2.2% were rated as warning, indicating a low level of visible artifacts. This outlines the fundamental difference between general- and clinical cohorts, where Slipsager et al. (Slipsager et al., 2020) report 2.0% of images to be non-diagnostic and 7.9% of images to be of decreased clinical interpretability. These numbers likely understate the difference in image quality, since raters in the Rhineland Study look for any type of small image artifact, that can be caused by subtle movement, while clinicians primarily focus on the brain areas and motion levels that are relevant to diagnosis.

To address the challenge of quantifying barely visible motion artifacts, a variety of metrics are derived as continuous scores from images or motion traces. *Image-based scores* (Callaghan et al., 2015; Esteban et al., 2017; Pannetier et al., 2016) derive measures of quality directly from the MR images, often without reference. While they capture motion artifacts in constrained settings of motion correction experiments (Callaghan et al., 2015; Pannetier et al., 2016), image based scores struggle to disentangle the causes of image quality and thus cannot reliably quantify motion, constraining their usefulness to correct the biasing effect of motion in statistical models. The challenge to directly estimate motion from an image is rooted in the complex interaction of head-motion and modern MRI acquisition protocols, which is, for example, affected by parallel imaging and the chosen k-space trajectories. Therefore, even metrics that are known to be correlated with motion for specific acquisition protocols and in specific cohorts (Zaca et al., 2018) are unlikely to generalize across all acquisitions, especially when motion levels may differ drastically between previously investigated clinical cohorts with tremor and general population cohorts. The advent of deep learning may offer an opportunity to capture the complex interactions between motion and acquisition, however, initial research is still limited to predicting the experts sentiment (Fantini et al., 2018); (Küstner et al. (2018a,b); Largent et al., 2021; Lei et al., 2022; Ma et al., 2020; Stpień et al., 2021; Sujit et al., 2019; Zukić et al., 2022), or perceptual quality metrics, based on simulated data (Sciarra et al., 2022). In initial work we leverage the motion quantification pipeline presented here with continuous motion scores to alleviate the reliance on manual labels (Pollak et al., 2023).

Since *motion-based scores* are computed from motion traces, they inherently reflect the participant’s motion unlike image-based scores. However, not all motion maps to image quality in the same way. Particularly, a line of work on prospective motion correction (Castella et al., 2018; Todd et al., 2015) proposes a metric, that calculates a weighted sum of head speed over planes of 2D MRI acquisition. The weights are determined by a numerical simulation of “impact” on image and show that for the investigated acquisition, motion at scan start or end is less detrimental to quality. Unfortunately the metric can not be generated for 3D MRI acquisitions and it is unclear how accurate the numerical simulation and definition of motion relate to image quality in real-world settings. In fact, the analysis of timing, i.e. when motion occurs, as well as motion patterns, e.g. breathing, abrupt motion or drift remains an open question.

### 1.4. Challenges

The field of within-scan motion tracking has yet to unify metrics and methods to associate head motion with human characteristics and image quality for retrospective analysis. Comparison of tracking method is challenging due to the absence of real-world ground truth motion measurements in most datasets.

We aim to establish an accurate and reliable within-scanner motion tracking method in the Rhineland Study (Breteler et al., 2014; Stöcker, 2016), an ongoing large population-based cohort study with mostly healthy participants. Multiple measures are taken to reduce head motion during the image acquisition, leading to overall low head-motion compared to clinical datasets, or datasets recorded with induced motion, where motion levels are markedly increased. For this purpose we rely on a high frequency markerless registration approach.

For reliable tracking of rapid and short motion events, multiple samples should be taken during the event. Since quick head adjustments or coughing can occur in a fraction of a second (Dbouk and Drikakis, 2020; Eberhardt and Topka, 2017), tracking frequency should be significantly faster. We recommend tracking speeds of more than 5 Hz. Slow methods with frequency of 2 Hz or less are unlikely to capture such events, since movement occurs either during recording of a single sampling point or between two recordings. Additionally, high sensitivity is required to quantify small movements, which are predominant in the mostly healthy cohort under investigation. Further, in a high-throughput environment, such as the Rhineland Study, we require a protocol with minimal setup to reduce the time participants spend in the MRI scanner. Ultimately, real-time performance is desirable to enable applications beyond retrospective data analysis, such as providing immediate feedback to operators during acquisition to repeat severely motion-affected scans, or for automatic online motion correction.

### 1.5. Contributions

In order to address these challenges, we propose a method for generating motion traces from depth images and aggregating them into a motion score. This pipeline consists of a dedicated robust registration, that generates motion traces and further post-processing steps, where traces are synchronized, smoothed, re-sampled and aggregated to yield a score of average motion. We outline this process in Fig. 2, where we show the building blocks of our processing pipeline as well as the method validation.

For the first time, we validate an optical tracking method on a large general population cohort with respect to three different indirect measures of head motion: fMRI motion traces (rigid transformations), respiration signal (scalar), and T1-weighted image quality measures. The introduced metrics can be applied to any within-scanner head tracking method and provide scalar scores to benchmark tracking performance.

Using these tests, we show that i) our method captures accurate motion traces, that are similar to motion traces from an established fMRI motion estimation toolbox, ii) our method captures high-frequency, respiratory signals as recorded on the participants chest, and iii) our motion score – from depth images during structural image acquisition – correlates with known structural image quality measures. We outperform the vendor-supplied registration method for markerless tracking on all three benchmarks. Furthermore, iv) we replicate previous findings of correlation between within-scanner head motion with age and body mass index, to demonstrate the applicability in the neuroimaging research domain. Finally, v) we show longitudinal, within-session effects of increasing motion with time (within sequences and overall scan time).

## 2. Materials and methods

### 2.1. Data acquisition

The data is acquired in the Rhineland Study (Breteler et al., 2014; Stöcker, 2016), a large population cohort study. The study invites participants from the area of Bonn, Germany, of age 30 and over and it is carried out in accordance with the recommendations of the International Council for Harmonisation’s “Good Clinical Practice ” standards (ICH-GCP). Written informed consent was obtained from all participants according to the Declaration of Helsinki. Approval was granted by the Ethics Committee of University Bonn.

During the Rhineland Study’s 1-hour MRI session, the scanner acquires up to 7 different MRI sequences, including T1-weighted (T1w) images and fMRI. Simultaneous optical head motion- and respiration tracking is performed throughout the session.

The participants are scanned with a 3T Siemens MAGNETOM Prisma MRI scanner (Siemens Healthcare, Erlangen, Germany) equipped with a 64-channel head-neck coil. The T1w images have a 0.8 mm isotropic voxel size and are acquired using a multi-echo magnetization-prepared rapid gradient-echo (ME-MPRAGE) sequence (van der Kouwe et al., 2008) with 2D acceleration (Brenner et al., 2014) and elliptical sampling (Mugler III, 2014) (repetition time (TR) = 2560 ms, inversion time (TI) = 1100ms, flip angle = 7°, field of view (FOV) = 256 × 256 mm, voxel size = 0.8 mm isotropic). The rapid whole-brain resting-state fMRI acquisition (Stirnberg et al., 2017) consists of 1070 frames with a 2.4mm isotopic resolution, each captured in 0.53 ms (TR = 530 ms, TE = 30 ms, flip angle = 16°, FOV = 192 × 192 × 144 mm, voxel size = 2.4 mm isotropic).

In one of the Rhineland Study’s scan centers, an infrared time-of-flight camera (TracInnovations, Copenhagen, Denmark) (Olesen et al. (2010, 2012); Slipsager et al., 2019), mounted on the head-coil of the scanner, captures depth images of a portion of the participant’s face during the scan. The depth images (also represented as point clouds) give a 3D representation of part of the participants face, captured through the opening between head-coil and a mirror in front of the participant’s eyes. While the camera position is adjustable on a rail in the superiorinferior direction for a participant-specific field of view, neither distance to the participant nor the left-right position can be changed. Therefore, captured facial regions vary depending on head-size and position.

**Fig. 1.**
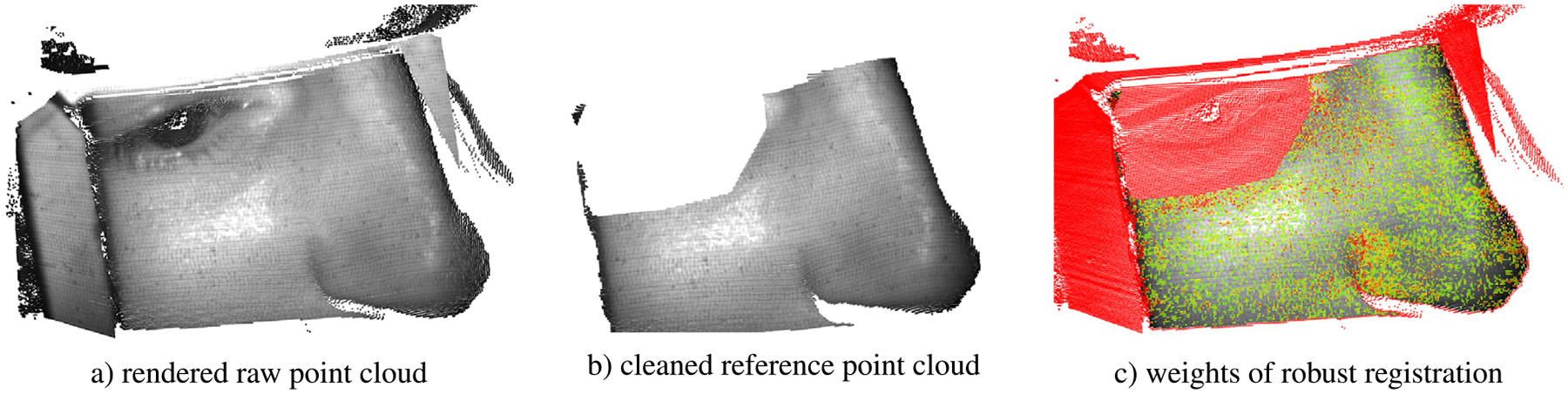
Our method registers frames of the depth-image video (**a**) to the reference depth-image captured prior to acquisition (**b**). Our robust registration employs point-wise registration weights (**c**) to ensure less reliable depth-values impact the registration less (yellow) or not at all (red). These unreliable regions are predominantly located around the eye and on the head-coil, but also in areas of high noise and non-rigid motion.

**Fig. 2.**
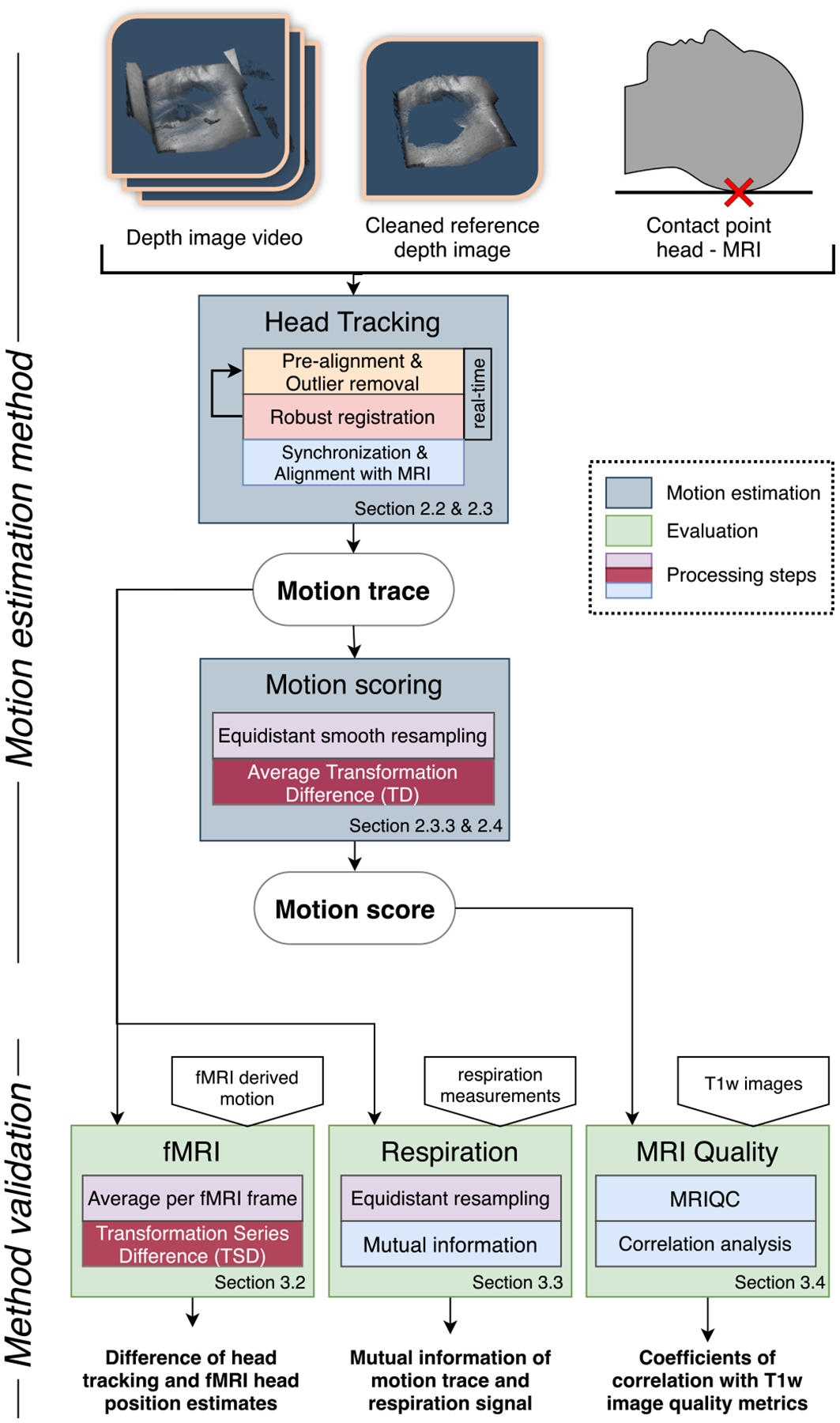
Overview of the proposed method and pipeline to derive motion scores from depth images of the face. The two outputs of our method are i) a motion trace (i.e. series of head positions), describing head motion during the scan session and ii) a per-sequence summary motion score.

Following the camera adjustment, the MRI operator defines a mask around the eye of the participant by drawing a polygon on a 2D grayscale image also captured by the motion camera. Additionally, TracSuite (the camera vendor software) (Bergström and Edlund, 2014) assists the operator in excluding areas unsuitable for the measurement of head motion (e.g. the head-coil). Fig. 1b) illustrates the cleaned reference point cloud resulting from this process. Throughout the session, the camera acquires depth-images at non-equidistant time points (approx. 32 frames per second), which are converted to point clouds using the camera’s field of view. Due to storage limitations, we only save every fourth point cloud, resulting in a sampling rate of approx. 8 Hz. The vendor software TracSuite has functionality for real-time head tracking. In addition to the raw-point clouds we also save the head motion estimates of TracSuite for method comparison, as well as time-stamps and metadata.

The Rhineland Study also measures the respiration of participants during the whole MRI session with a respiration sensor (PERU 098, Siemens Healthineers, Erlangen), that rests on the participants chest and captures its movement.

To reduce the head motion during the scan session, MRI operators ensure that:

1. The head is stabilized by inflatable (preferred), or foam cushions.
2. Each session is interrupted by two short, scheduled breaks during which the operator speaks with the participant in the scanner.
3. Nature scenes are shown during the whole session (Greene et al., 2018; Huijbers et al., 2017; Madan, 2018; Vanderwal et al., 2015), except for the initial resting state functional MRI scan, when participants are asked to fixate a cross on the screen.
4. Participants are instructed to remain still and stay awake.

The fraction of sessions with simultaneously acquired motion data (at the time of preparation of this work) consists of a total of 573 participants with a male/female distribution (self-reported sex) of 266/207 and age-range from 30 to 95 years. For developing and optimizing registration variants, we randomly select 10 participants, whose data we exclude from the method comparisons. For downstream analysis, we exclude 79 participants that did not complete the whole protocol or where motion data is not available for all scans (yielding 494 cases).

### 2.2. Motion estimation method

We compute a motion trace, by rigidly registering all point clouds (generated from depth images) to the manually cleaned reference point cloud. The result of the registration is the mapping of all captured point clouds to the reference, giving information how much the participants head moved during the scan. A classic solution for point-to-point registration problems is the Iterative-Closest-Point (ICP) algorithm (Arun et al., 1987). To address accuracy and robustness concerns of face registration such as noise and non-rigid facial skin motion, we introduce a robust criterion (following (Bergström and Edlund, 2014)) as well as a regularizer.

#### 2.2.1. Robust registration

We formulate the head tracking task as a sequential, rigid registration of point clouds captured during the scan session *P*_*k*_ *ϵ P*_*1…N*_ to the reference point cloud *P*_ref_. The resulting rigid body transformations consist of a rotation matrix $R_{k}\in {\mathbb{R}}^{3\times 3}$, with $R_{k}^{T}R_{k}=I$ and det (*R*_*k*_) = 1, and a translation vector $t_{k}\in {\mathbb{R}}^{3}$. We call a time series of these rigid transformations (rotation and translation) a motion trace. The registration of *P*_*k*_ → *P*_ref_ can be characterized by solving the optimization problem:

(1) $$R_{k},t_{k}=\underset{R_{k},t_{k}}{\text{arg min}}\sum_{p_{k}\in P_{k}}\psi \underset{≔{\text{res}}_{p}}{\underset{︸}{\left(\underset{p_{\text{ref }}\in P_{\text{ref }}}{\text{min}}{\left\|R_{k}p_{k}+t_{k}-p_{\text{ref }}\right\|}_{2}^{2}\right)+\lambda }}.$$

In contrast to the unmodified ICP algorithm, where *ψ* (res _*p*_) is the identity function, we compare different robust criterion functions and, furthermore, introduce a regularizer *λ* = *λ*(*R*_*k*_, *t*_*k*_) to discourage implausible solutions.

#### 2.2.2. Robust criterion

Numerous candidates for the robust criterion *ψ*(res _*p*_) exist, such as Tukey Bi-weight-, Cauchy-, Welsch-, and Huber functions (Bergström and Edlund, 2014), which all down-weight or disregard point correspondences that do not satisfy the rigidity assumption based on the distance res_*p*_ to the nearest neighbour in the reference. We compare the criterion functions in Section 3.1 below and choose Huber’s function as the best performer:

(2) $$\psi \left({\text{res}}_{p}\right)=\{\begin{array}{cc} \frac{1}{2}{\text{res}}_{p}{\;}^{2} & \text{if }{\text{ res}}_{p}\leq Z\\ Z\;{\text{res}}_{p}-\frac{Z^{2}}{2} & \text{if }{\text{ res}}_{p}>Z \end{array}\;Z=\left(1+r_{b}\right)\text{median}\left({\text{res}}_{1\ldots N}\right),$$

where the hyper-parameter *r*_*b*_ determines a relative threshold beyond which the impact of large residuals is reduced and res _*p*_ is the residual associated with point *p* **∈** *P*. This robust approach also offers a solution to the partial registration problem, where a part of the registration object may be visible in one point cloud, but not in the other. While outliers in *P*_ref_ would never be considered due to the correspondence search min_*P* ref_ ∈^*P*^_ref_, the robust criterion reduces the effect of outliers in *P*_1…*N*_ by assigning them weights close to zero.

#### 2.2.3. Regularization

In addition to the robustness criterion, we aim to incorporate known mechanics about within-scanner head motion into the registration. Because the camera only records a part of the face visible through the head-coil, the whole head pose is inferred based on features in the limited field of view of the camera at the front of the head (as seen in Fig. 1). This can reduce registration accuracy at the back of the head, where the skull is farthest from the visible region. Given that the head is resting on the pillow and tight padding prevents it from sliding, the contact point of the pillow and the back of the head can be encouraged to act as an additional (soft) anchor. Visual inspection of fMRI sequences confirmed that the back of the head is more stable than other regions, which is consistent with previous findings (Frost et al., 2019), and motivates this additional regularization.

The regularizing point *P*_*ref*_ at the back of the head is added to the optimization as a per-participant constant. We weigh the anchor with a weight of *α* = 0.03 (a fraction fixed across all participants determined by experiments in Section 3.1) and multiply by the sum of all weights *Ψ* (res_*p*_), resulting in the term:

(3) $$\lambda \left(R_{k},t_{k}\right)=\alpha \left(\sum_{p_{k}\in P_{k}}\psi \left({\text{res }}_{p_{k}}\right)\right)\left(R_{k}p_{\text{reg }}+t_{k}-p_{\text{reg }}\right)$$

with additional variables in analogy to Eq. 1. We determine *P*_*ref*_, by first aligning the T1w image with a template (with FreeSurfer’s robust register tool (Reuter et al., 2010)) and by then searching for the first voxels in the anterior direction. To get a reliable estimate, we ignore background noise by robustly conforming intensities and find the voxel closest to the median center of the cluster to use as the point *P ref*. Finally, the regularizing point is mapped into the coordinate system of the motion camera as described in Section 2.3.1.

#### 2.2.4. Performance and robustness considerations

To enable fast and robust sequential registration, we add two pre-processing steps, for each point cloud. These steps leverage the temporal consistency of the sequential point cloud data.

First, when registering the point cloud of the current frame to the reference, we initialize the rigid transformation with the result of the previous registration. This provides a good starting point for the registration optimizer and we only need to fine-tune the registration for the small movement possible within the sampling rate of the camera (approximately 125 ms in our case). Thus, the optimizer can converge with relatively few iterations, resulting in quick computation and additional robustness against unrealistically large movements (e.g. caused by measurement errors in a single frame).

Second, we discard distant outlier points prior to the optimizer loop. This is helpful, because shifts of head position can decrease the size of the overlapping region between the current and the reference point cloud. Such distant outlier points do not aid the registration, but affect the robust criterion through the median residual (see Eq. 2). We address this, by discarding distant outliers farther than 5mm from the reference point cloud, which enables us to keep the robust bound constant throughout all registrations. The registration accuracy is not affected, as the 5 mm distance threshold is conservatively estimated (about 30 standard deviations of mean motion in 125 ms). In addition, the a-priori removal speeds-up the overall computation.

Finally, to further increase registration speed, we under-sample the raw point clouds with a factor of three. This undersampling reduces head pose estimation time to approximately 110 ms, which is faster than the sampling interval of 125 ms per point cloud.

### 2.3. Post-processing

The time points inherited from point cloud samples, cause our motion traces to be non-equidistant in time and not always accurately synchronized with the MRI scanner’s internal clock. To facilitate the use of motion estimates in downstream analyses, we establish a post-processing pipeline that synchronizes all acquired MR images with the output transformations, reduces noise, and re-samples the transformations.

#### 2.3.1. Aligning motion-tracker and MRI space

In order to compare head motion traces (e.g. camera-based traces with fMRI traces), they need to be defined with respect to a common coordinate system. Here, we choose the coordinate system of the participant’s T1-weighted image. Since we can easily approximate the head center (and size) from the T1w image, the aforementioned mapping also enables us to accurately quantify motion within the head volume and to find the back of the head for regularization. To get the mapping between both coordinate spaces, we register the surface through the first high-intensity voxels of the T1w-image in posterior direction with the reference point cloud captured by the motion camera. To ensure good initial alignment, we pre-align the T1w images to a standardized template with FreeSurfer’s robust register tool (Reuter et al., 2010).

The established correspondence between reference point cloud and the participant-specific anatomical space (T1w image) ties our motion tracking pipeline into common neuroimaging toolboxes, like FreeSurfer (Fischl, 2012). For example, registering functional MRI images to the T1w image with FreeSurfer’s bbregister (Greve and Fischl, 2009) enables a comparison of fMRI-based motion traces and motion traces from our method in a common space.

#### 2.3.2. Time synchronization

The motion camera software (Slipsager et al., 2019) annotates individual point clouds with timestamps relative to the MRI session by requesting the time from scanner ad-hoc and correcting for latency. To address imperfect clock synchronization, we search for the offset (max. 15 seconds) that yields minimal differences between our and the fMRI motion traces (see Section 2.4.3). After synchronizing scanner and motion-tracking timestamps, our post-processing pipeline automatically reads meta-data from the scanner’s DICOM files and annotates point clouds accordingly with the corresponding sequence and participant codes.

#### 2.3.3. Re-sampling transformations

The time-stamps associated with the head poses determined by our registration are not equidistant, since the tracking system provides measurements at irregular intervals. Because post-processing steps, like motion scoring, require equidistant measurements at varying frequencies, we implement a re-sampling directly on the translation vectors and quaternions (Gramkow, 2001) of the motion trace by averaging over a sliding window with a triangular weight function. To parameterize this step for all applications we specify the window size and triangle slope separately, with a slope of zero resulting in an average along the window size (e.g. for averaging head poses during the acquisition of an fMRI frame).

### 2.4. Motion metrics

After determining the motion traces during a scan session and performing post-processing to prepare them for down-stream analysis, we present multiple summary metrics to describe head motion in method validation and statistical analysis.

#### 2.4.1. Head pose difference

For method validation and downstream analysis we require a metric that compares two head poses. While previous work compares head poses by translational and rotational errors of the head poses (Laustsen et al., 2022; Pardoe et al., 2021), we aim to combine head rotation and translation into a single score that weights both components according to the medical imaging application, as suggested by Wilke (2012, 2014).

Here, we employ the root mean square deviation for transformations (Jenkinson, 1999) (further *head pose difference*, HPD) to reduce the six degrees of freedom of a rigid transform (3 rotational and 3 translational) between two head poses into a single distance score. HPD is derived by averaging the displacements within a ball, which represents the head (Jenkinson, 1999). These displacements reflect the movement from one pose to another (described by transformation matrices). We approximate the parameters to this metric (head radius and location) from the population-average (distance from face to head center: 82.5 mm) and the individually placed scan origin, respectively. In practice, the latter is defined by the technician as part of the image acquisition for each participant.

The HPD distance measure is applied in two different contexts: 1. to quantify motion, i.e. to quantify how much the head pose changes between two measurements, and 2. to compare two transformations, e.g. for a comparison of methods.

#### 2.4.2. Motion score

In contrast to pairwise pose differences, we also desire a robust summary motion score during a time interval (further *motion score* in millimeter per second), e.g., to quantify average motion for each MR sequence and include it into statistical analysis.

The proposed *motion score* is calculated by:

1. Re-sampling the motion trace (triangle slope = 0.1, window size = 9 samples), to create equidistant motion traces with head poses at exactly 8 Hz and to reduce measurement noise (see Section 2.3.3 Re-sampling).
2. Calculating the *head pose difference* between consecutive head poses and summing these differences for each one second interval (see previous section).
3. Averaging the one second *head pose differences* during the time of a single MR sequence.

The resulting summary scores are used as measure of average head volume displacement throughout each MR sequence.

#### 2.4.3. Motion trace difference

For method validation, we require a metric that compares two motion traces (series of head poses). When one wants to compare two motion traces calculated from the same data (same sampling points), the *head pose difference* can be used on each pair directly. When comparing two motion traces acquired in the same time interval, but based on different samples and methods, we need to assert that

1. sampling time points agree,
2. transformations use the same coordinate space, and
3. comparisons are independent of the specific reference that was used in each method.

We ensure the first requirement of matching sampling points, by using the previously established re-sampling method. Second, we ensure a common coordinate space in the anatomical space of the structural MR scan as described in Section 2.3.1. The third challenge arises because head poses are always defined in comparison to a specific reference pose and these references can differ across methods (e.g. captured at different times, such as the beginning of the scan session or the beginning of the fMRI sequence). We, therefore, introduce a new metric *motion trace difference* (MTD) to compare motion traces from fMRI and optical tracking, that is independent of the specific reference pose. Thus, we compute transformations between each pair of poses for each method and average the HPD distance between corresponding transformations across methods (see Appendix D for details). This symmetric metric enables a fair comparison between two motion traces from different methods, with a summary measure of average displacement in the head.

In this work, we compare our motion traces to fMRI based motion traces, which are commonly used for in-MRI head motion analyses (Beyer et al., 2020; Dosenbach et al., 2017; Huijbers et al., 2017; Pardoe et al., 2016; Savalia et al., 2017). While fMRI-based registrations are not without errors, we expect them to capture low-frequency head motion sufficiently well, especially since the whole skull is utilized for registration instead of only an area of the face as for the motion camera. We estimate the motion from fMRI with MCFLIRT (Jenkinson et al., 2002) and compare MCFLIRT’s motion trace to our’s by first averaging our motion trace over the length of the fMRI frame (570 ms). Then, we map both traces to the reference space and calculate the MTD as described above. While we consider all 1070 fMRI frames per participant in final results, 70 equally spaced fMRI frames sufficiently describe the performance characteristics for method development and testing in Section 3.1.

#### 2.4.4. Mutual information of head motion and respiration

For additional sensitivity validation of head tracking methods, we aim to investigate how much of the densely sampled (256 Hz) respiration signal is contained in the head motion trace. The scalar respiration signal is measured by a respiration sensor attached to each participant’s chest and indicates the respiratory phase and amplitude in the units of the sensor. As pre-processing, we re-sample the motion trace with our interpolation (see Section 2.3.3, triangle slope = 0.1 *s*^−1^ and window size = 3 samples) and down-sample the respiration signal, with linear interpolation, to the point cloud measurement frequency of 8 Hz. Next, we compare the scalar respiration signal to motion traces with six degrees of freedom. We accomplish this by converting the transformation matrices of the motion trace into two vectors for translation and rotation (the latter being represented by quaternions). We ensure, that no sign flip occurs during conversion, by testing that the dot product of rotations in quaternion representation is positive and use all four components of the quaternion representation. The concatenation of both vectors results in a motion signal over time, that can be compared to a scalar respiration measurement. Finally, we estimate mutual information (Kraskov et al., 2004; Pedregosa et al., 2011) between the respiration signal and each of the motion signals. The sum of mutual information scores then represents how much information captured by the respiration sensor is contained in the motion trace.

### 2.5. Statistical methods

We require statistical tests for the validation of our methods as well as for the analysis of cross-sectional and longitudinal motion effects in a scan session. First, we perform a paired difference test across methods using the non-parametric Wilcoxon’s signed-rank test. Second, to test whether two measures are linearly correlated, we use Pearson’s-r (e.g. for comparison of motion with image quality metrics). For both tests we employ the implementations of the SciPy (Virtanen et al., 2020) Python library. To reproduce previous cross-sectional results, which show correlation of risk factors age and body mass index with motion, we use a least squares regression model with BMI, age, and sex as dependent variable and average motion over all scan sequences as the variable of interest.

Finally we test the hypothesis, that participant motion increases over the course of the scan session. For this task, we model motion using a longitudinal linear mixed effects model (LME). We include time as well as interaction terms of time with age, sex, and BMI. The model is described by the term (participant *i*, sampling point *j* at every 1 second interval):

(4) $$Y_{i,j}=\beta_{1}+\beta_{2}t_{i,j}+\beta_{3}\;{\text{BMI}}_{i}+\beta_{4}\;{\text{age}}_{i}+\beta_{5}\;{\text{sex}}_{i}+\beta_{6}t_{i,j}\;{\text{BMI}}_{i}+\beta_{7}t_{i,j}\;{\text{age}}_{i}+\beta_{8}t_{i,j}\;{\text{sex}}_{i}+b_{i}+e_{i,j}$$

with *bi* as subject specific random intercept, *β*_1_ as global intercept and e _*i*_ the random error. The dependent variable *Y*_*i,j*_ is motion (i.e. head speed) in (mm/s). t _*i*_ is the time from start of first image acquisition, BMI is the body mass index, and age the participant’s age in years. The latter two markers are centered around zero prior to model fit. We also include sex as a categorical variable. Data points are only sampled during scan sequences and not during breaks, where larger motion can occur. Both statistical models are implemented in the statsmodels Python library (Seabold and Perktold, 2010).

## 3. Results

In this section we compare our method to the state-of-the-art registration – the software of the motion cameras manufacturer (TracSuite (Slipsager et al., 2019, 2022), Version 3.0.0). We determine both methods’ quality by measuring similarity to functional MRI motion and to respiratory traces. Further, we explore the correlation of motion scores with structural image quality measures as well as with known correlates of motion (BMI and age). These downstream correlation analyses serve as a validation experiment for the pipeline consisting of the registration method and the proposed motion score for statistical analysis. Finally, we apply our method in the Rhineland Study to investigate, whether participants move more in later MR scans during a scan protocol and whether fMRI motion estimates can be used as an approximation of motion for later sequences.

### 3.1. Ablation study

Prior to the comparison with another method, we peform an ablation study. This type of study investigates performance of a system by removing, adding, or exchanging individual components one-at-a-time to understand the contribution of each component to the overall system. Here, we investigate which choices are most important for the final method performance by running different variations of the robust registration and thus determine our final method for the subsequent analyses. To prevent leaking information about the test data into method development we use a separate set of 10 scan sessions for the optimization of our method and exclude these cases from subsequent evaluation.

**Fig. 3.**
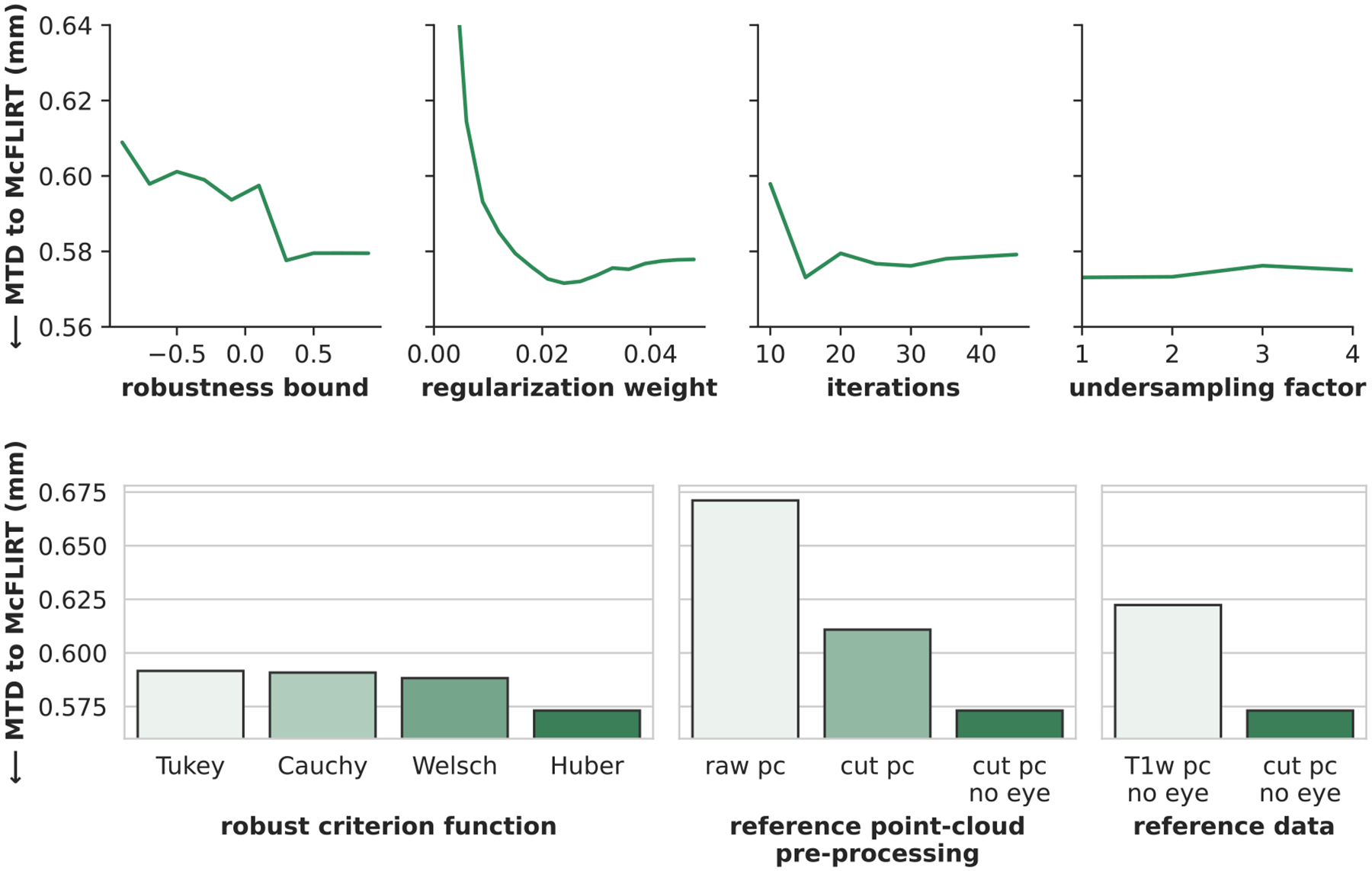
We test different configurations of our registration method in an ablation, using the transformation series difference (MTD, see Section 2.4.3, lower is better) to a fMRI based motion traces of MCFLIRT as metric. The top row depicts results for continuous parameters of the registration (left to right): the robust bound *r*_*b*_, relative weight of regularization, the number of iterations of the robust registration, and the under-sampling factor of the input point clouds on a grid. The bottom row shows four different robustness criterion functions (Bergström and Edlund, 2014) (left), and four different reference point clouds (right) including a raw point cloud (*raw pc*), a point cloud generated from the T1w image with the eye cropped out (*T1w pc no eye*), the cleaned point cloud with cropped out head-coil and smoothing (*cut pc*), and the cleaned point cloud where the eye is additionally cropped out (*cut pc no eye*).

The MTDs to MCFLIRT’s motion traces (i.e. the difference to fMRI-based motion estimates) of the registration variations are shown in Fig. 3). We investigate the impact of the robust bound *r*_*b*_, as well as the criterion function - both of which define the robustness of the iterative closest point registration. We find the best criterion function is Huber’s function with *r*_*b*_ = 0. 2. We test three other criterion functions, but for the same, optimized robust bound and same asymptotic variance of the functions, the difference in performance is small. A noticeable performance increase, however, occurs for the regularizer, whose introduction reduces the transformation series difference to the fMRI scan by more than 0.5 mm.

To show the trade-off between method speed and performance we vary the number of internal iterations of the robust iterative closest point algorithm. We find that there is no performance gain for more than 15 performed iterations, indicating fast convergence for most registrations. We conservatively choose 30 optimizer iterations for the final method. We also observe little performance loss for an under-sampling of point clouds on a grid up to every fourth point. This specific comparison, however, may be insensitive to random noise, due to the averaging of multiple motion camera based head poses for the comparison with the low-frequency fMRI based motion trace. In an additional evaluation with the respiration signal on the ablation data set (no dedicated Figure), we find that under-sampling decreases performance when looking at mutual information with respiratory measurements (1: 0.79, 2: 0.77, 3: 0.76, 4: 0.71; [undersampling factor: mutual information]) showcasing the importance of different secondary metrics for method validation. Since MI drops substantially for an under-sampling factor greater than three, we choose three as factor for the final method – which still allows for online registration.

Finally, we investigate how the reference point cloud affects the registration performance. We compare different pre-processing steps of the reference point cloud and find that omitting pre-processing (*raw pc*) reduces registration performance. Both smoothing and removing artifacts as a pre-processing (*cut pc*) as well as the additional manual masking step (*cut pc no eye*) increase accuracy. As an additional experiment, we extract a reference point cloud from the T1w image, which contains the whole face (*T1w pc no eye*). In our case, this MR-based reference performs worse than cleaned references based on the depth camera data. The full view of the face may, however, prove to be an advantage for large motion cases, where overlap of visible face region with the camera reference could become too small.

### 3.2. Comparison to fMRI motion estimates

As a first method validation, we compare the multiple kinds of motion traces from the depth camera to motion traces based on resting state fMRI. The evaluation follows the same setup as our ablation study in the previous section, but is performed on the large dataset of 563 fMRI acquisitions.

**Fig. 4.**
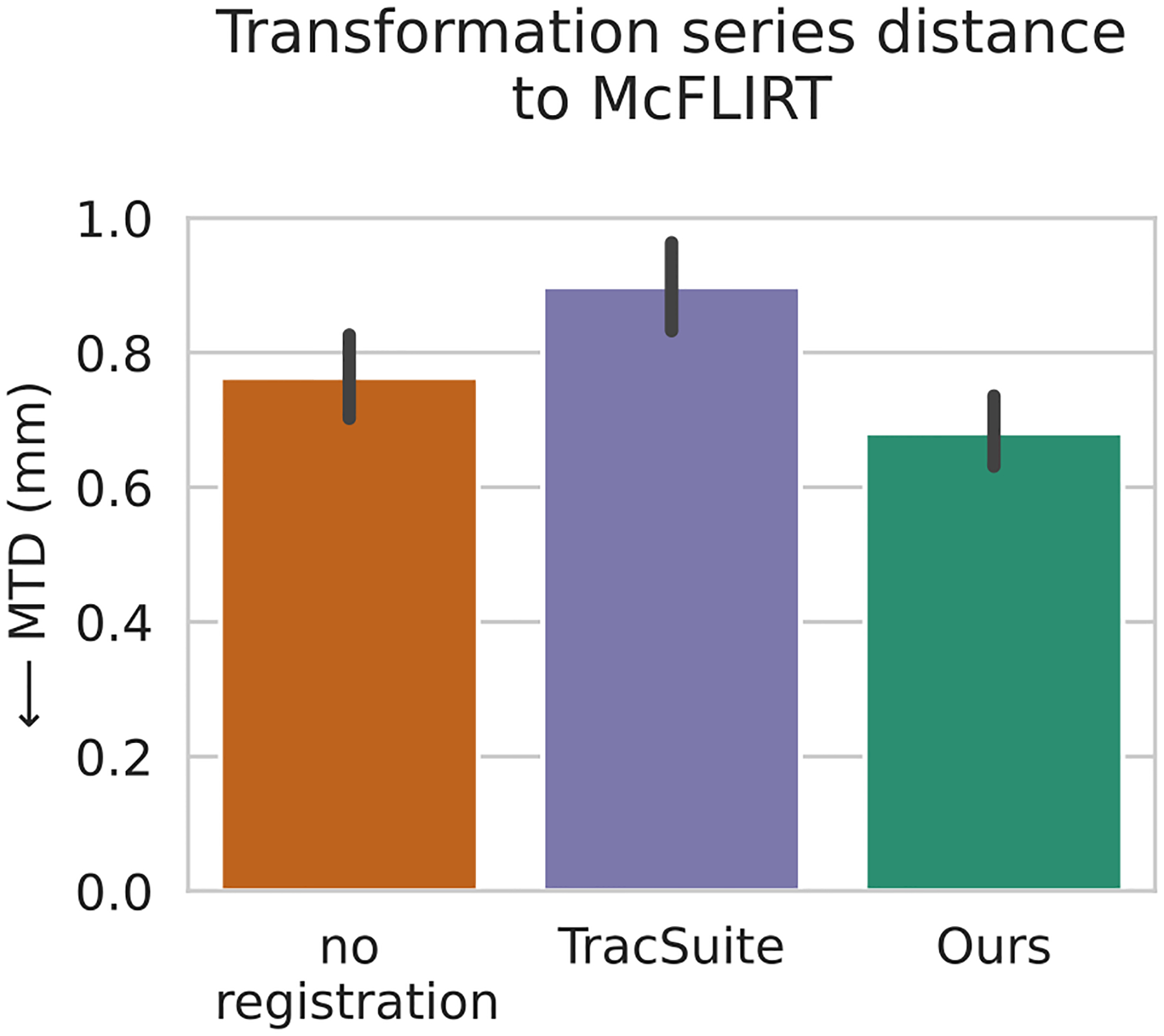
Comparison of registration methods with fMRI motion traces as reference standard. The y-axis shows the transformation series difference (MTD) to motion traces of MCFLIRT. “no registration ” describes a motion trace, where zero motion is estimated (identity matrices). All pairwise differences between methods are significant, measured by the Wilcoxon signed-rank test (*p* <.001).

The three evaluated methods are: 1. The trivial “no registration” method, which only predicts a series of identity matrices, 2. TracSuite – the camera manufacturers method (Slipsager et al., 2019), and 3. our registration method. We show the results of the analysis in Fig. 4. Our method is significantly closer to the fMRI motion estimates than TracSuite or assuming no motion (*p* < .001). This shows that our method captures similar movements as MCFLIRT from the independent fMRI acquisition. Surprisingly, TracSuite’s estimates are significantly farther from fMRI based motion traces for the investigated population cohort than assuming no motion (*p* < .001).

### 3.3. Comparison to respiratory chest movements

We estimate the mutual information of motion traces and measurements of a respiration sensor on the participants chest. This comparison showcases the ability to capture subtle head movements, that can also be measured by a high-frequency respiration sensor. We test the mutual information between respiratory chest movements and 1. a random motion baseline (6 independent uniformly distributed random variables between 0 and 1), 2. TracSuite, and 3. our registration.

**Fig. 5.**
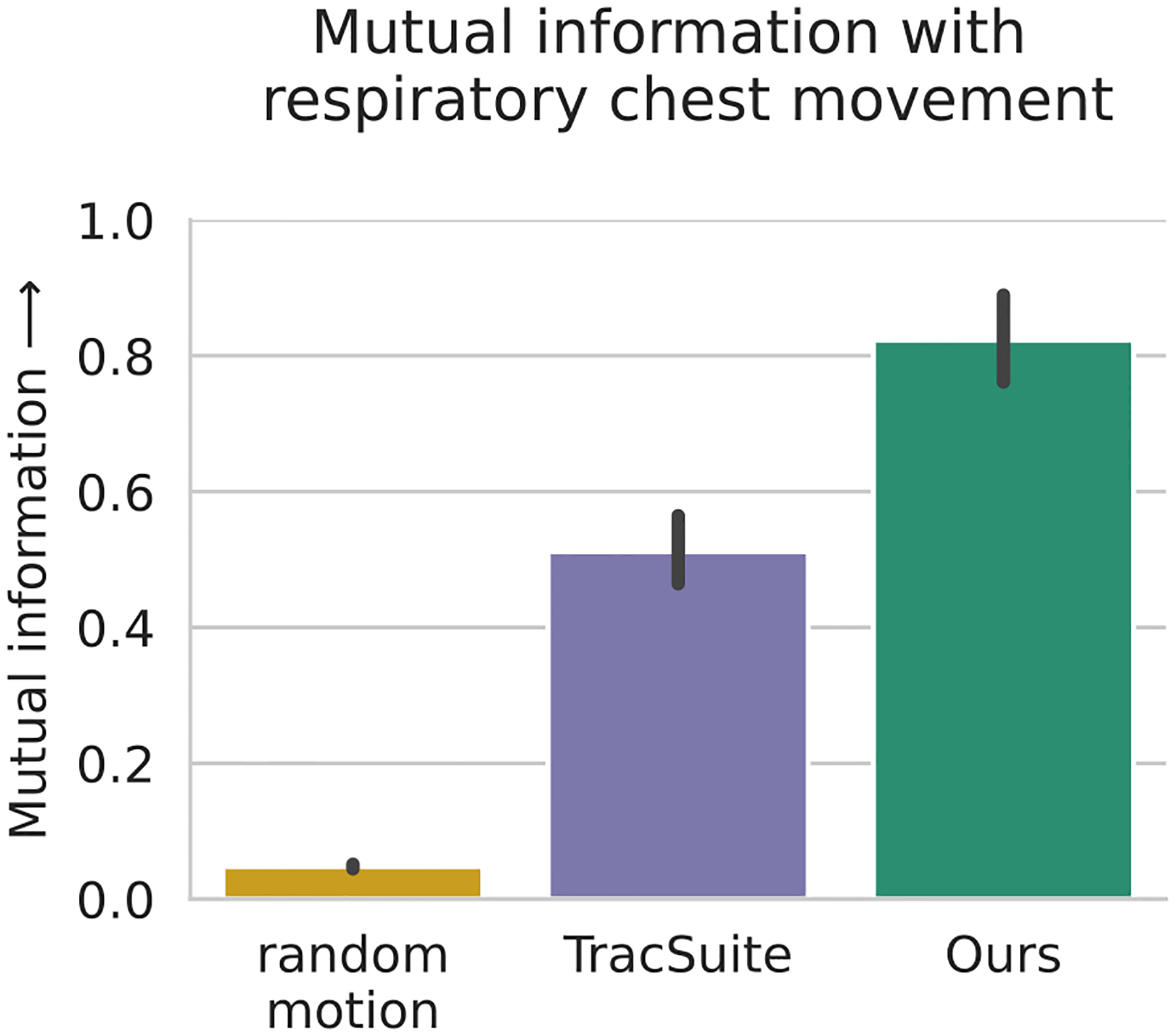
Comparison of registration methods by mutual information (MI) with respiratory chest sensor movements. High MI indicates that chest movements are contained in the head motion traces. Random motion describes a randomly generated transformation series. All pairwise differences between methods are significant, measured by the Wilcoxon signed-rank test (*p* <.001).

The mutual information between respiration- and motion measurements is shown in Fig. 5. Both head tracking methods capture information about the participant’s breathing and chest movements. Our method significantly outperforms TracSuite in this validation (*p* <.001). For additional qualitative comparison, we show the motion traces and respiration signal of one representative sample in Appendix B.

### 3.4. Motion correlates with image quality metrics

To jointly validate our registration as well as our motion scoring method, we test the correlation of *motion scores* for the T1w image acquisition with different measures of structural image quality.

**Fig. 6.**
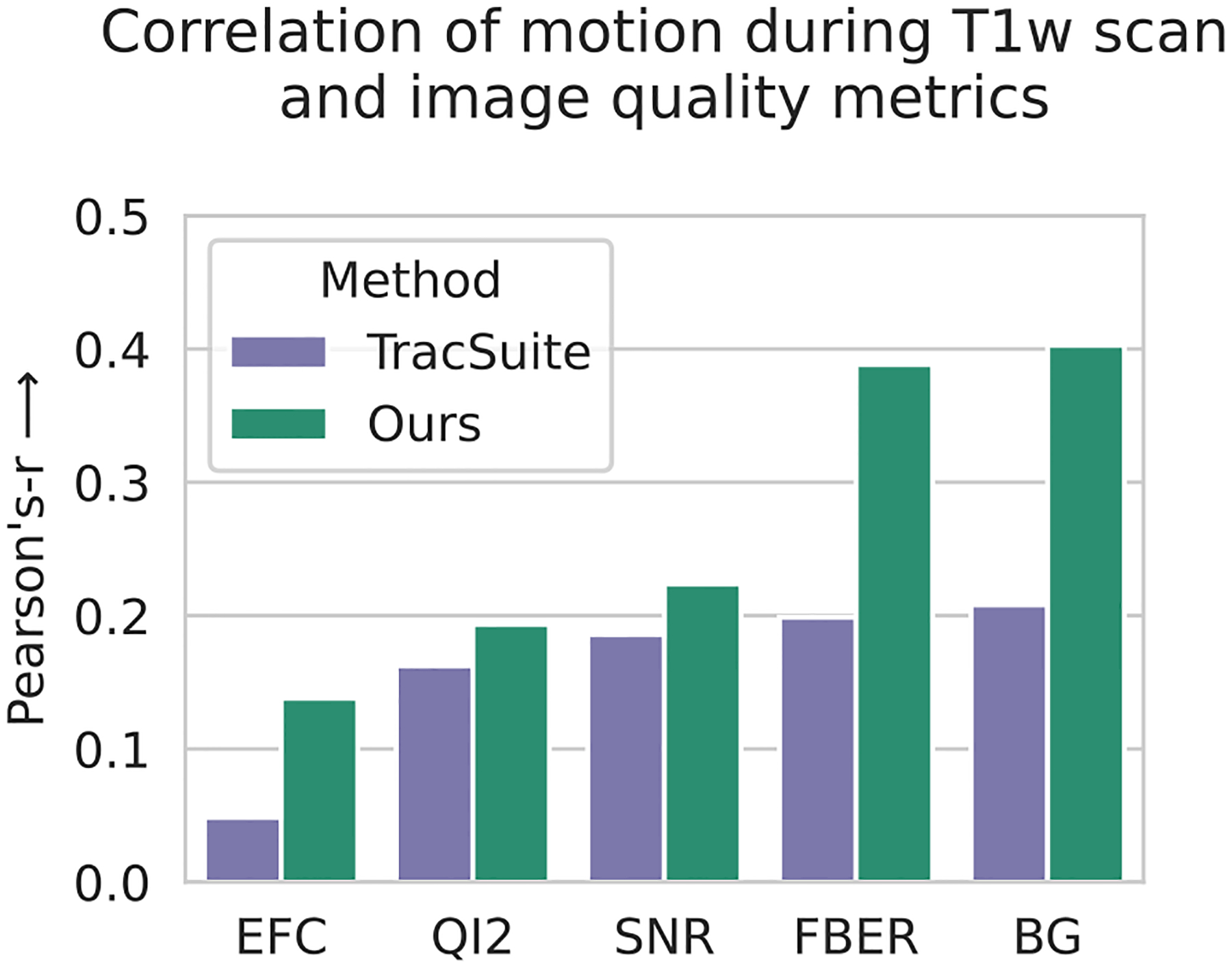
Comparison of registration methods by linear correlation coefficient (Pearson’s-r) of calculated motion score and T1w image quality metrics. Since competing methods do not generate a motion average, we use our motion score to aggregate both motion traces. The shown metrics generated by the MRIQC (Esteban et al., 2017) toolbox are: the entropy focus criterion (EFC) (Atkinson et al., 1997), Mortamet’s quality index 2 (QI2) (Mortamet et al., 2009), signal to noise ratio (SNR), foreground-to-background energy ratio (FBER) (Shehzad et al., 2015), and mean background intensity (BG).

The image quality metrics are determined with the MRIQC (Esteban et al., 2017) toolbox and have been either associated with motion (Atkinson et al., 1997; Provins et al., 2023; Shehzad et al., 2015) or with perceived reduction in quality (Mortamet et al., 2009; Shehzad et al., 2015). Since the competing method, TracSuite, does not provide a scoring method, we apply our scoring method to it’s motion traces. The results are presented in Fig. 6, where the pair-wise linear correlation coefficient (Pearson’s-r) is shown for five quality metrics, that are associated with motion and motion artifacts in structural images. The motion score based on our registration method has a higher correlation than the score based on TracSuite across all metrics.

### 3.5. Motion correlates with BMI and age

As a final validation, we test whether we can find known correlates of head motion with the combination of our registration method and motion scoring. Increased head motion is known to be associated with increased body mass index (BMI) (Beyer et al., 2017, 2020; Couvy-Duchesne et al., 2014; Ekhtiari et al., 2019; Hodgson et al., 2017; Kong et al., 2014) and with age (Andrews-Hanna et al., 2007; Chan et al., 2014; Madan, 2018; Pardoe et al., 2016; Savalia et al., 2017). To test whether we can replicate these cross-sectional effects with motion measured by our method, we fit a linear regression model with average motion across all scans in the session as the dependent variable and the independent variables BMI, age, and sex. The correlation of both age (*r* = 0.0023, *p* <.001) and BMI (*r* = 0.0053, *p* <.001) with participant motion is highly significant (see Fig. 8), confirming previous findings with our method. Participants sex is not significantly correlated with in-scanner head motion (*r* = 0.0089 *, p* =.446). These expected findings showcase, that our metric can be used to quantify motion for downstream analysis.

### 3.6. Longitudinal motion effects during a scan session

Finally, we apply our method by modeling motion effects and interactions over the course of the whole scan session. Since the optical tracking method is independent of the scanner we can, for the first time, directly compare motion in a 1h scan session across multiple MR sequences. We perform this analysis with the linear mixed effects model (LME) described in Section 2.5.

**Fig. 7.**
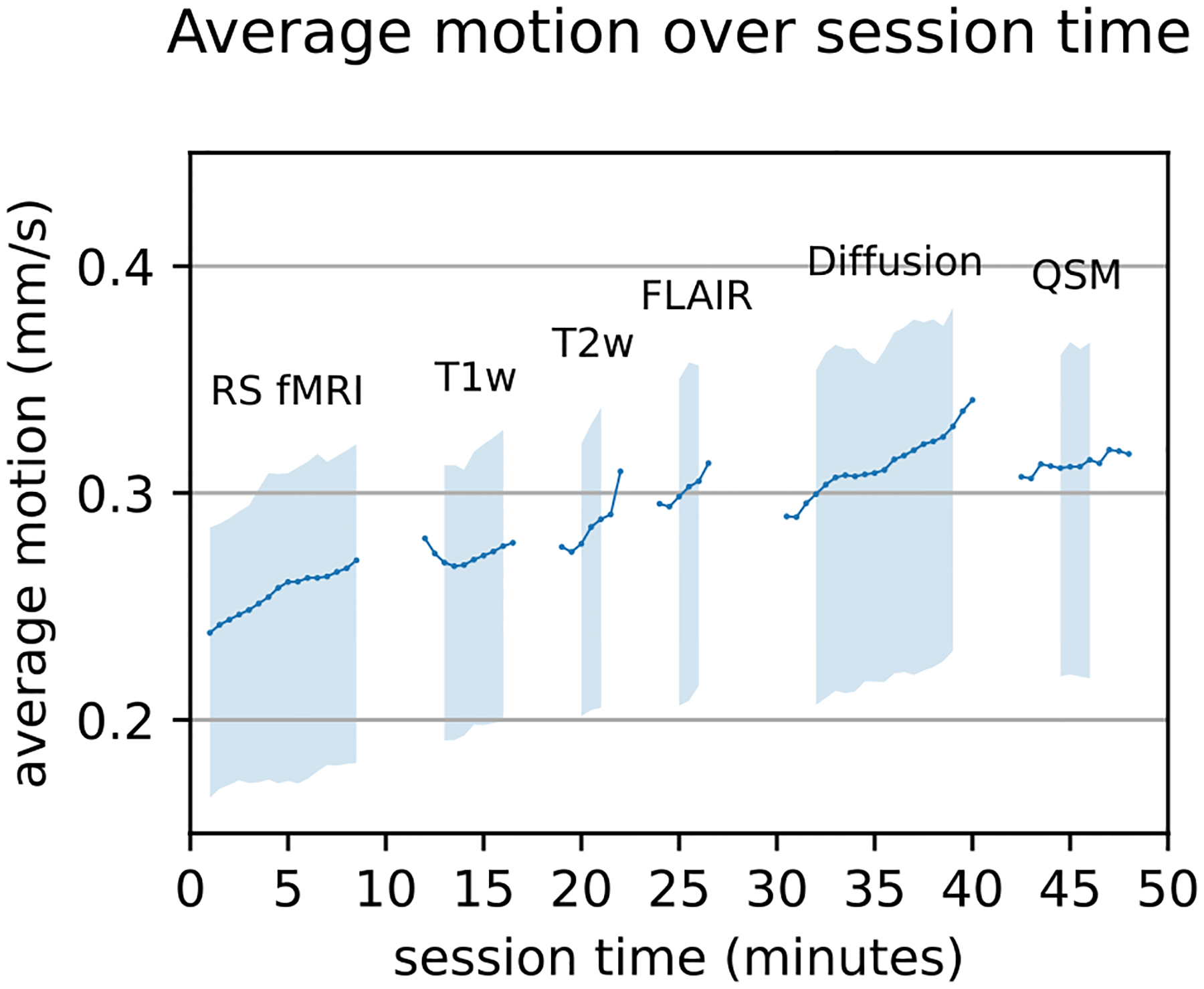
Motion (30-seconds moving average) increases during image acquisition and throughout the full MRI session. The line indicates the average of participants motion, if samples of more than 20% of participants are available and the shaded area indicates the interquartile range if samples of more than 80% of participants are available. The number of available samples differs over time due to varying gaps between sequences. Annotations indicate the different MR sequences.

**Fig. 8.**
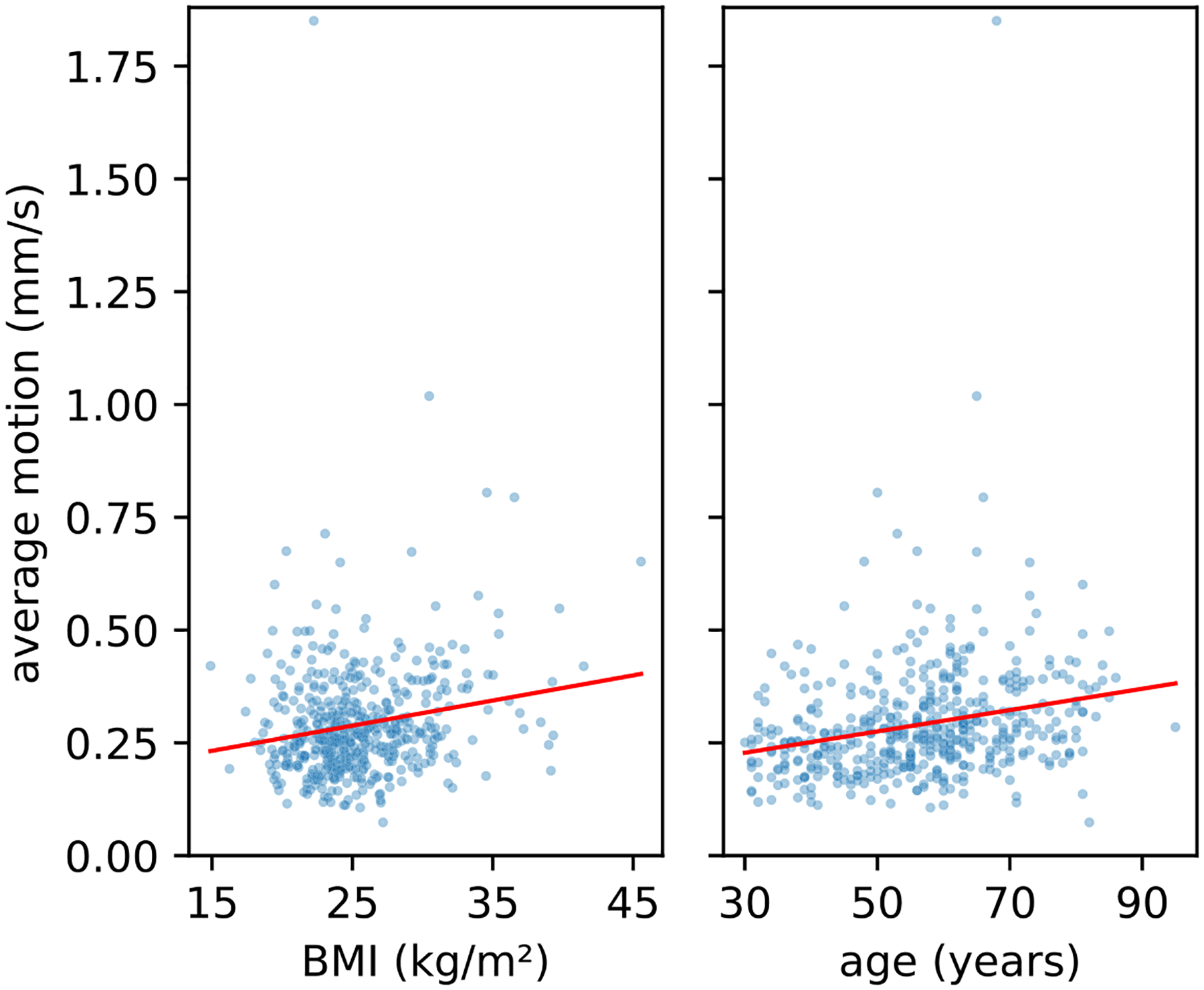
Our average motion score correlates significantly with body mass index (BMI) and age in a linear correlation model, that includes sex, BMI, and age (*p* <.001). Depicted is the scatter data with independent linear fits.

In the standardized protocol of the Rhineland Study, motion increases significantly over time with approximately 0.6% per minute (0.001751 mm/s) of mean motion across all participants [MMAAP] (time: *p* <.001 *, z* = 706.145) as illustrated in Fig. 7. A similar increase can be observed per one year of participant age (0.7% MMAAP,0.002077 mm/s, *p* <.001, = 5.159). We also find a strong correlation with the body mass index (BMI), where one point of BMI increased motion levels by around 1.5% MMAAP (0.004397 mm/s, *p* <.001, *z* = 3.411). There is no significant difference in motion levels across sexes (*z* = 0.493 *, p* =.622).

The linear mixed effects model also contains time interactions. We observe a significantly higher increase of motion over time for increased age and body mass index of participants (time × BMI: 0.021% MMAAP 0.000060 mm/s, *z* = 132. 906 , *p* < . 001, time × age: 0.002% MMAAP, 0.000002 *z* = 40. 322 , *p* < . 001). This indicates that levels do not only increase in groups of higher BMI and age, but that the increase of motion over time in the scanner is amplified by these correlates of head motion, though the additional increase is very small in comparison to the general increase of motion during the session. Participant sex has a larger measured impact on motion increase over time with male participants having a slower increase of motion over time (time × sex: 0.069% MMAAP, 0.000199 mm/s, *z* = 52. 392 , *p* < . 001), despite no significant cross-sectional effect. All of the described interaction effects, however, are weak compared to the overall increase over time, suggesting that cross-sectional motion differences are similar throughout the scan sessions.

**Fig. 9.**
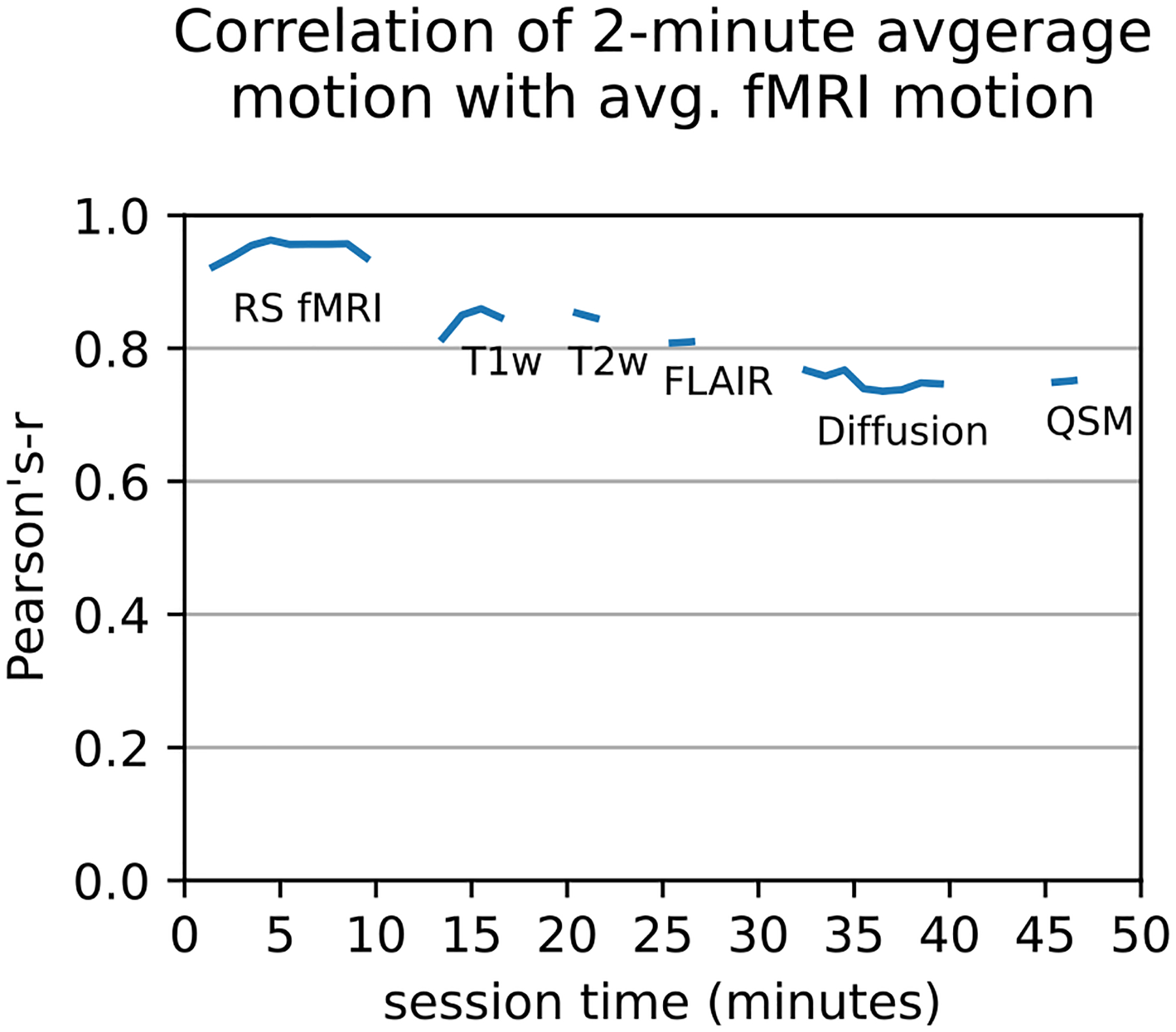
Paired linear correlation (Pearson’s-r) analysis: motion during a resting state fMRI (RS fMRI) sequence is a good approximation for depth camera motion estimates (1-minute moving averages) during following sequences. The measures were averaged in 1 minute bins and bins with less than 90% of total available samples were discarded. For a description of sequences and correlations per image acquisition see A.1.

Since within-scanner motion tracking is not always available, we test whether motion can be inferred from one sequence to later sequences. A previously proposed motion correction strategy is using readily available motion traces from fMRI to exclude participants with increased motion levels (Pardoe et al., 2016; Savalia et al., 2017) from morphological statistical analyses in order to reduce bias. In the Rhineland Study’s standardized protocol the resting state acquisition is first in the session. For the first time, we compare direct estimates of motion during functional MRI and other MRI sequences, to see whether motion levels are stable throughout the scan session. In a paired linear correlation analysis, shown in Fig. 9, we observe a high correlation coefficient for all scans in the one hour protocol (Pearson’s-r; adjacent to fMRI: *t* = 0. 88 , *p* < . 001; 35 minutes after fMRI: *t* = 0. 77 , *p* < . 001).

## 4. Discussion

We present a robust method to extract motion traces and a representative motion score from a depth video of the face. The proposed registration method outperforms the vendor-supplied method on three indirect validation tasks. Besides the registration method we introduce tools to compare head motion traces. We, furthermore, perform association studies of head motion as a variable of interest with BMI, sex, age and scan session time in the Rhineland study.

The proposed registration method combines a robust point-to-point registration with a-priori outlier detection and a regularizer specific to the face tracking application. A key to the performance and robustness of our method is exploiting the high sampling frequency of the camera. Contrary to e.g. fMRI based motion tracking, the movement performed between measurements is much smaller, allowing for a detailed estimation of the head motion. This also allows a highly constrained registration, which targets a smaller range of movements possible in 125 ms and permits real time processing. Markerless tracking, however, comes with a trade-off. Contrary to MRI-based motion tracking, only a small part of the head is visible and the skin can cause non-rigid movements. We address these limitations with two additions to the registration. To address non-rigid movements and noise on the measured face area we add a robustness criterion (Bergström and Edlund, 2014) and to achieve stable inference of the head position at the back of the head, we introduce an anatomically motivated regularizer. We evaluate the impact of these additions and their parameters in the ablations study (Fig. 3) and see large improvements for sensible robustness criteria and regularization.

Direct method validation with ground truth motion is exceedingly rare and difficult in the head motion tracking domain. A promising avenue for this are movable head phantoms (Einspänner et al., 2022). However, these phantoms are not widely available and can not yet imitate realistic movement patterns of participants. Therefore secondary metrics of head motion, like fMRI-based estimates should be chosen for indirect method validation. We believe that multiple secondary metrics are required for method validation in order to reduce bias in the comparison of two imperfect motion estimates. Unfortunately, this practice is currently not widespread, with some previous markerless and marker-based optical tracking methods only using MRI motion correction capabilities as real-world validation (Maclaren et al., 2012; Slipsager et al., 2019). With the proposed extension of Jenkinson’s transformation difference to motion traces and the use of three secondary sources of motion measurements: 1. fMRI-based motion traces, 2. respiratory chest movement, 3. structural image quality metrics, we hope to contribute to the development and validation of head tracking methods in general.

A limitation of our test setup is the storage space for motion sequences, which only allows us to save every fourth point cloud for retrospective registration. The vendor-supplied software ”TracSuite ” processes all point clouds directly upon capture and, therefore, has access to additional point clouds. We expect this asymmetry of conditions in the method comparison with otherwise same conditions to favor Trac-Suite, since more point clouds can be used to reduce the acquisition noise (which was a key challenge throughout the development of this method). Future applications of our method executed in parallel with the MR acquisition could also benefit from the additional temporal resolution. Furthermore, it would allow: i) using our metrics and accurate tracking to provide real-time feedback about participant motion to MRI operators, and ii) improving previously proposed (Frost et al., 2019; Slipsager et al., 2022) markerless online motion correction methods.

Our method outperforms TracSuite on all three validations. While TracSuite has high mutual information with respiratory chest movement, matching the findings of (Slipsager et al., 2019), it performs worse than no registration (identity) in comparison to fMRI based estimates for our 563 participant dataset. These inconsistent results underline the importance of multiple different metrics when performing indirect method validation. The surprising under-performance of TracSuite might be an indicator that it targets high-motion, clinical groups and does not generalize well to compliant participants with little head motion, where no motion is a decent assumption. Since the competing method is not open source, we cannot further investigate this result. The overall low motion in our dataset can be seen by the seemingly high performance of the “no registration baseline ”. Participants remain still for large parts of the scan causing the residual MTD to MCFLIRT of the baseline and our method to be of similar magnitude. At short times, the difference between the two methods, can be larger than the head motion. However, when averaging over the whole dataset, our method agrees with MCFLIRTs estimates of head position. To showcase the difference to high-motion datasets we show the comparison to fMRI for a case of extreme, induced motion in Appendix C, where both methods clearly outperform the baseline. A strength of our methods is that the fMRI validation can be performed without additional hardware and therefore our method can easily be reoptimized and validated for different scan setups and cohorts. For the extreme motion case, for example, we re-ran the optimization and found that regularization is not helpful in large motion settings, but exchanging the motion-tracker derived reference by the reference derived from the T1w MR-image improves the registration performance for this case, which has very different, larger patterns of movement, due to induced motion and removed padding.

While the estimation of head positions is optimized with fMRI-based estimates as ground truth, our score of average motion is theoretically motivated. In this work, we do not consider the way motion impacts MRI scans differently depending on the type and time-point of motion. Nonetheless, we can show correlation with image quality metrics and achieve higher correlation coefficients than the competing method in a fair comparison (using the same scoring). This implies that our method quantifies motion relevant for acquisition more accurately than the other methods.

The strength of the proposed scoring is the direct application as scanner-independent metric for statistical analyses in population studies. We show this by performing a straightforward statistical analysis to replicate previous findings of motion associations with BMI and age and, furthermore, apply our method to test whether motion increases throughout the scan sessions. We find that time in the scanner is indeed a strong, significant correlate of motion, confirming previous fMRI-based studies of in-scanner head motion (Meissner et al., 2020). Motion is, for example, increased by approximately 29% after 35 minutes of scanning in our dataset. This trend can also be observed in Fig. 7, where we show motion increases over time. A confounder of this visualization is a reduced sample size at the start and end of MRI sequences. Since breaks between scans are inevitably of different length, the start and end of each sequence include fewer samples, containing participants with exceptionally short or long breaks. We outline this effect, by removing the confidence interval and plotted line for fewer than 20% and 80%, respectively. The resulting graph shows an intra-scan and intra-session increase of motion, but an inter-scan reduction of motion in most cases, which is in agreement with the previously demonstrated effectiveness of short breaks (Meissner et al., 2020). We also note, that motion measurements seems to increase more than expected during the Diffusion weighted image (DWI) acquisition and decreases afterwards. This phenomenon might be connected to the rapidly changing gradients during DWI, which cause loud noises and vibrations. This, in turn, could affect participants’ head motion levels and possibly also the measurement system itself. We, however, did not find any noticeable vibration artifacts in the motion tracking point cloud data by visual inspection.

Despite a marked increase of motion over time, overall motion levels can be considered low. The Rhineland Study with its compliant and predominantly healthy participants incorporates several procedures to reduce head motion, which results in small absolute effect sizes. This is corroborated by high structural image quality (shown by the afore-mentioned low number of WARN or FAIL ratings compared to clinical cohorts) and the fact that “no motion ” is a decent estimate for fMRI based head motion traces, while the difference is higher by an order of magnitude in an induced motion experiment (see Appendix C).

Nevertheless, the LME also reconfirms significant cross-sectional associations of BMI and age with motion. Given that motion can bias downstream morphometric analyses (Alexander-Bloch et al., 2016; Reuter et al., 2015), it is likely beneficial to control for head motion, even when it is small, to disentangle motion effects from real anatomical changes. The age correlation further underlines that quantifying and controlling for head motion could be important when aiming to find subtle longitudinal differences.

Age and BMI are only two of the many potential markers that correlate with motion. We did not yet investigate any disease states, smoking, sleep, sport, or medications, which is an obvious avenue for future work. Even beyond, high-frequency motion tracking can be used to analyze motion patterns of participant groups. Previous research, for example, has shown causation between increased BMI and increased motion levels (Beyer et al., 2020) with fMRI based motion estimates, but it remains unclear what type of movements were exhibited by overweight participants. The authors cite alterations in the respiratory system (Littleton, 2012) and alterations in dopaminergic signaling (Tomasi and Volkow, 2013) as possible reasons for a decrease of head motion after drastic weight loss, due to bariatric surgery. As we show in our validation, the proposed method captures head motion associated with respiration, in addition to larger, impulsive movements. By measuring the change in these different motion types, researchers could gain valuable information in the possible mechanisms of increased head motion.

We note, that sex is not significantly associated with motion in the investigated population, despite previously shown significant differences, with females moving less than males (Huijbers et al., 2017), especially for pediatric patients (Dosenbach et al., 2017; Frew et al., 2022; Madan, 2018; Pardoe et al., 2016). Surprisingly, we find that males’ motion levels increase significantly less than females’ motion levels during the scan session. We also find significant interactions between time in the scanner with BMI and age, with increased BMI and age associated with a faster increase of motion over time. These results indicate, that the potentially increasing discomfort is amplified in older participants and participants with larger BMI. The interaction effect sizes, however, are very small compared to the cross-sectional motion effects and the overall motion increase with time.

The small size of interaction effects indicates a high rank-consistency of motion levels during the scan session as previously shown during multiple functional MRI measurements (Savalia et al., 2017). Since motion tracking is not readily available on all sites, we investigate whether we can measure direct paired linear correlation between motion during the initial fMRI sequence and following sequences. High correlation indicates that a single measurement of motion at the beginning is predictive of motion levels, even at later stages of the scan protocol. Therefore an initial measurement could be enough to correct for motion in all sequences, given a consistent ordering of scans. Fig. 9 shows that correlation with average motion during resting state (RS) fMRI is highly correlated with two minute intervals during the same sequence, but the correlation drops sharply at the begin of the next sequence (T1w image acquisition) and then continuously decreases. This could be caused by a different scanning environment during functional scan (with a fixation cross, typical for RS fMRI) and other sequences where a movie is shown to participants. Watching movies has been shown to reduce head motion compared to a fixation task (Greene et al., 2018; Huijbers et al., 2017; Vanderwal et al., 2015), which might influence correlation of motion levels. Nonetheless, we see rather high paired linear correlation between average RS fMRI and the following sequences, indicating that, in absence of dedicated motion tracking, fMRI can be used to estimate motion levels during the whole scan session.

Due to the significant increase of motion over time, we recommend that important sequences or sequences with high sensitivity to motion should be acquired first - especially in long sessions. The common assumption of increased motion at the beginning of the first scan can be rejected in accordance with previous literature (Meissner et al., 2020). Further, to reduce motion bias, the order of sequence acquisition should ideally remain constant in longitudinal studies.

Overall, we introduced a performant, accurate method for robust registration of depth images of the face. This method, in combination with extensive validation and reliable aggregation into an average score, is capable of finding previously known and unknown motion effects in a large population cohort study. It, furthermore, provides a sensitive measure of head motion to analyze and control motion effects in statistical association studies.

## Figures and Tables

**Fig. B1.**
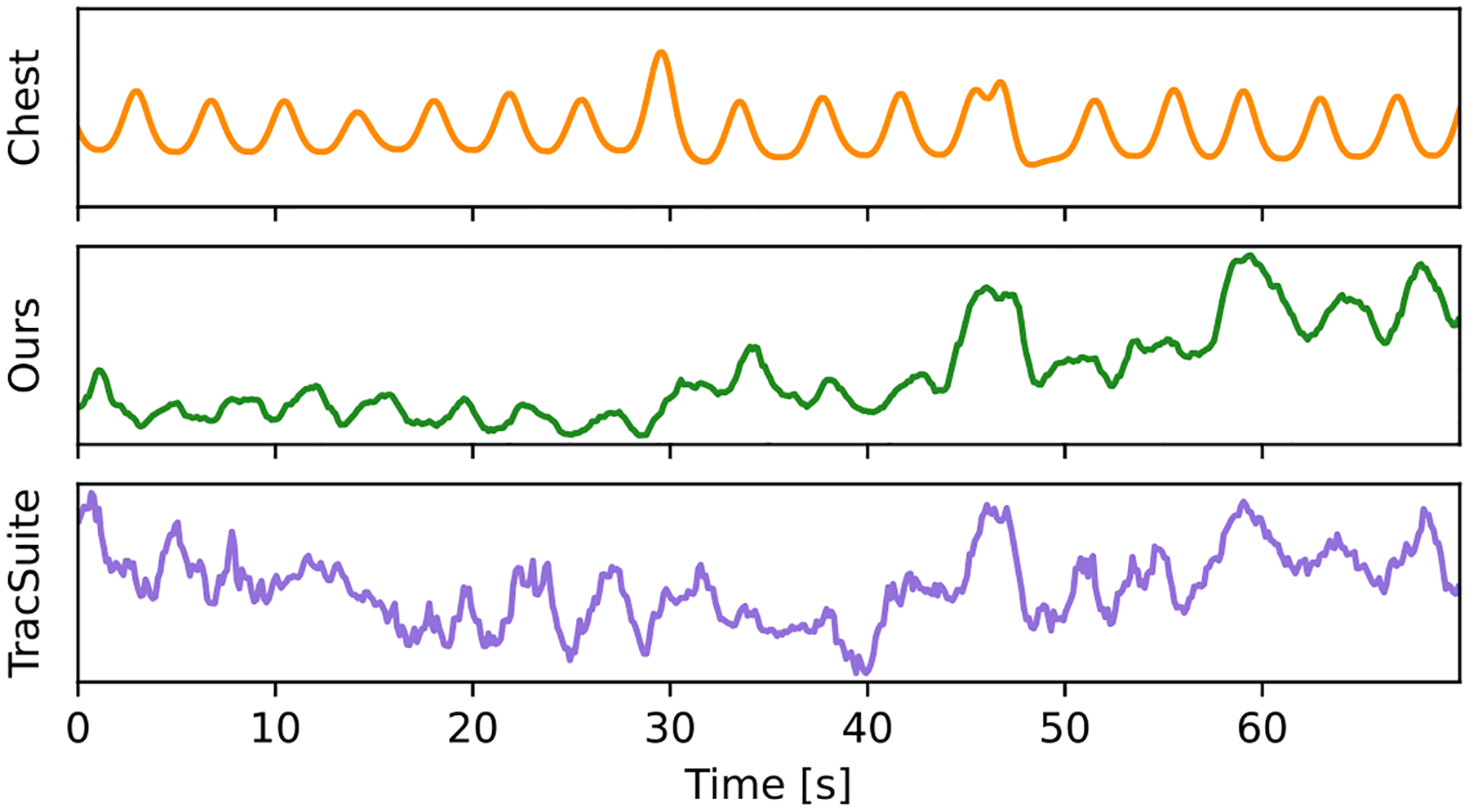
This figure shows a qualitative comparison of respiratory chest movements (top) with our registration method (middle) and TracSuite (bottom) for a short, representative time interval of a single participant.

**Fig. C1.**
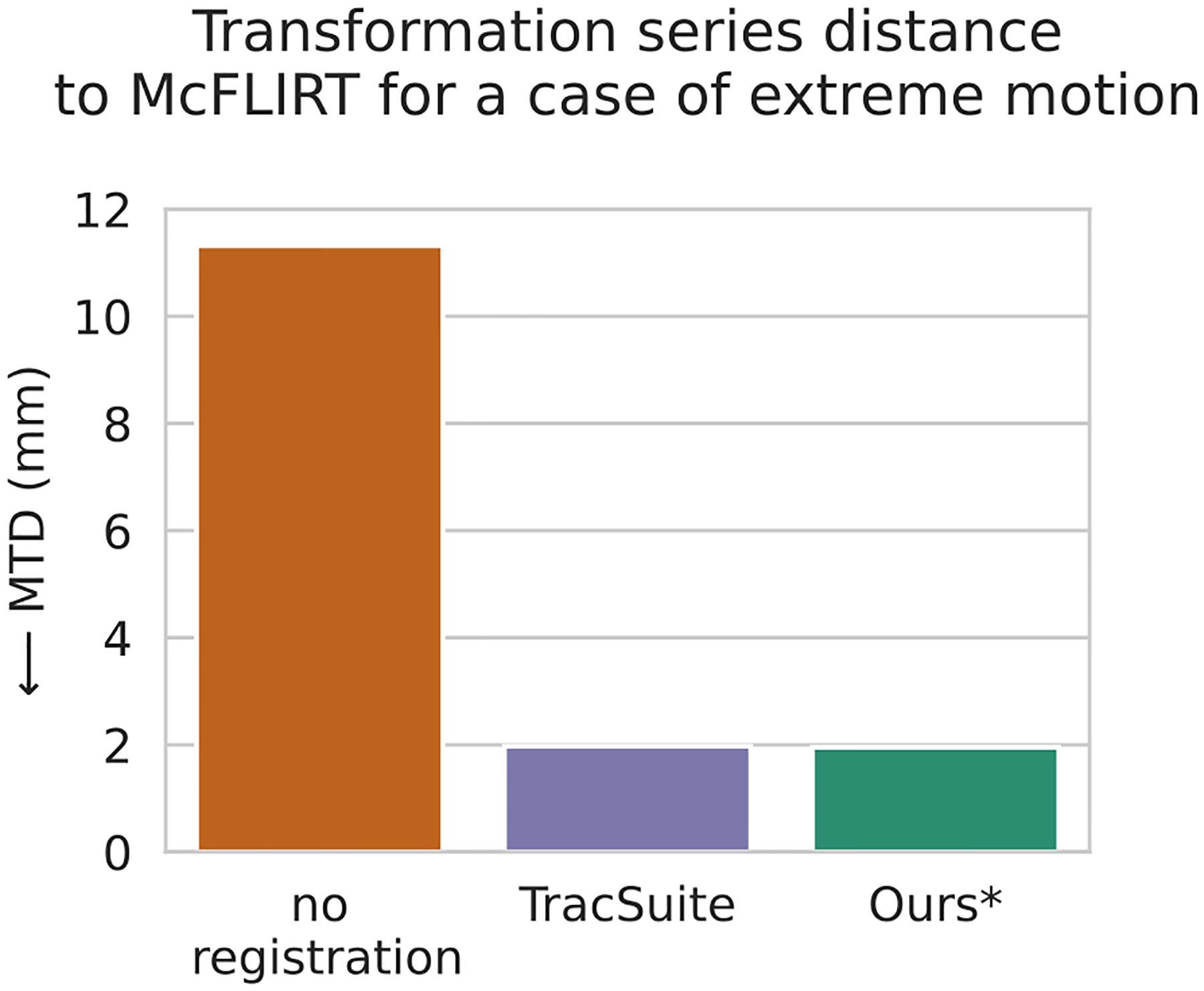
Comparison of registration methods with fMRI motion traces as reference standard (as Fig. 4) for one case of induced motion. “Ours*” is our method with adjusted parameters for higher motion levels.

## Data Availability

This work uses data from the Rhineland Study. Data of the Rhineland Study is not publicly available because of data protection regulations. However, access can be provided to scientists in accordance with the Rhineland Study’s Data Use and Access Policy. Requests to access the data should be directed to Dr. Monique Breteler at RS-DUAC@dzne.de. The source code for the registration and validation methods will be made public upon acceptance at https://github.com/Deep-MI/head-motion-tools.

## Appendix A. Average motion levels

**Table A1:** We report the average motion for all MR scans in the protocol, as defined by our motion score (see Section 2.3). Additionally we show the pair-wise linear correlation coefficient of motion measured during the resting state fMRI sequence and other sequences. Finally, we show the approximate length of each image acquisition in seconds.

Sequence	average motion [mn/s]	Pearson’s-r to fMRI	scan duration [s]
Resting state functional MRI	0.255 ± 0.126	1.000	625
T1-weighted imaging	0.273 ± 0.124	0.879	395
T2-weighted imaging	0.301 ± 0.162	0.863	287
Fluid-attenuated inversion recovery (FLAIR)	0.301 ± 0.153	0.824	277
Diffusion-weighted imaging	0.315 ± 0.147	0.768	682
Quantitative Susceptibility Mapping (QSM)	0.313 ± 0.148	0.775	380

## Appendix B. Qualitative comparison of respiration- and head motion signals

We show a qualitative visualization of the respiration signal (relative chest movement), TracSuite, and our method (motion quantified by HPD to initial reference depth image). Since TracSuite does not offer postprocessing, we use our smooth equidistant re-sampling method to denoise and re-sample both sequences equally. The result in Fig. B1 shows respiratory movements in both motion traces, however, the TracSuite signal contains a lot of ”jitter ”, which could indicate noisy measurements. This is also reflected in the mutual information scores (TracSuite: 0.40, Ours: 0.73), even though the case was chosen to be as close as possible to both methods’ 50th percentile mutual information score. At 45 seconds we can observe a distinct pattern of an elongated spike with two peaks in all three signals.

## Appendix C. Induced motion experiment

We show the comparison of fMRI-based motion estimates to our registration method and TracSuite for one case, where the participant was asked to move in the scanner with removed padding. This results in much higher motion levels, than in the Rhineland Study (Fig. 4), causing a larger difference between “no registration ” baseline and motion estimates of MCFLIRT. We use the fMRI frames to re-adjust our method for higher motion levels, as shown in Section 3.1, resulting in the parameters: reference point cloud = T1w MRI, regularizer weight = 0, max. optimizer steps = 50, *r*_*b*_ = −0.5, criterion function = Huber’s function. For this single recorded high-motion case the difference of methods is not significant. Results can be seen in Fig. C1 below.

## Appendix D. Motion trace difference

Here we describe the computation of motion trace differences in more detail. Prior to comparison we ensure with re-sampling and registration (see Section 2.3) that the *n* sampling time points of the two different motion traces agree and that they are defined with respect to the same aligned coordinate system (here from T1w MRI).

A motion trace consists of multiple head poses that can be described as homogeneous transformation matrices *M*_*i*_ = *P*_*ref*_→ *P*_*i*_ mapping a reference measurement (e.g. point cloud captured at the start of the session) *p*_*ref*_ to a measurement at time point *i* (*P*_*i*_) of the trace. For the second motion trace, this reference is usually collected at a different time point and, thus, transformations are not directly comparable. This could be solved by selecting the same time point as the reference for both traces, e.g. time point one *P*_*ref*_ = *P*_1_ and multiplying every transformation with the inverse transformation of the first time-point $P_{1}\to P_{1\ldots n}=M_{1\ldots n}\times M_{1}^{-1}$. However, fixing a single reference time point in this fashion introduces bias into the motion trace. When we consider *M*_1_ to consist of registration error *ϵ* and true head pose *T*, we see that calculating $P_{1}\to P_{1\ldots n}=M_{1\ldots n}\times M_{1}^{-1}=M_{1\ldots n}\times {\left(T\times \epsilon \right)}^{-1}$ prop agates the error 𝜖 to every head pose.

To avoid emphasizing a specific time point with unknown head pose error we rephrase the motion-traces with respect to all possible time points. Consequently, we calculate the MTD, by averaging the HPD of all *n* head poses with all possible *n* time points as the reference *p*_*i*_→ *p*_*j*_ with *i, j* ∈ 1.*n*. This results in the term:

(D.1)
$$\text{MTD}\left(A_{1\ldots n},B_{1\ldots n}\right)=\frac{\sum_{k=1}^{n}\sum_{l=1}^{n}\text{HPD}\left(A_{l}A_{k}^{-1},B_{l}B_{k}^{-1},h_{c},h_{r}\right)}{n^{2}}$$

to compare two motion traces *A*_1…*n*_, *B*_1…*n*_ (i.e. a series of homogeneous transformation matrices or head poses), with a head model of radius *h*_*r*_ = 82.5 mm and center *h*_*c*_. The resulting metric is a performant, symmetric, and interpretable measure of the difference between two transformation series, that describes the average difference of head pose estimates in millimeter.

## Appendix E. Ethics

The Rhineland Study is carried out in accordance with the recommendations of the International Council for Harmonisation’s “Good Clinical Practice ” standards (ICH-GCP). Written informed consent was obtained from all participants according to the Declaration of Helsinki. Approval was granted by the Ethics Committee of University Bonn.

The raw data visible in Figure 1 and 2 is specifically acquired for this work. The participant has given informed consent to inclusion of the motion tracking raw data in this publication.
